# The effect of Ingenol-B on the suppressive capacity of elite suppressor HIV-specific CD8+ T cells

**DOI:** 10.1371/journal.pone.0174516

**Published:** 2017-05-03

**Authors:** Abena K. Kwaa, Kennedy Goldsborough, Victoria E. Walker-Sperling, Luiz F. Pianowski, Lucio Gama, Joel N. Blankson

**Affiliations:** 1 Center for AIDS Research, Department of Medicine, Johns Hopkins University School of Medicine, Baltimore, Maryland, United States of America; 2 Department of Molecular and Comparative Medicine, Johns Hopkins University School of Medicine, Baltimore, Maryland, United States of America; 3 Kyolab, Campinas, Brazil; Massachusetts General Hospital, UNITED STATES

## Abstract

**Background:**

Some latency-reversing agents (LRAs) inhibit HIV-specific CD8+ T cell responses. In a prior study of protein kinase C (PKC) agonists, we found that bryostatin-1 inhibited elite controller/suppressor (ES) CD8+ T cell suppressive activity whereas prostratin had no effect. Ingenol-B is another PKC agonist with potent LRA activity both by itself and in combination with the bromodomain inhibitor JQ1; however its effect on CD8+ T cell mediated control of HIV-1 replication is unknown.

**Methods:**

CD8+ T cells were isolated from ES and treated with bryostatin-1, prostratin, ingenol-B, and JQ1 as well as a combination of each PKC-agonist with JQ1. The cells were then tested in the viral suppression assay. To assess possible mechanisms of inhibition, CD8+ T cells were treated with the LRAs and analyzed for the expression of various immune cell markers.

**Results:**

Ingenol-B had no effect on the ability of ES CD8+ T cells to suppress viral replication, however, the combination of ingenol-B and JQ1 caused a modest, but significant decrease in this suppressive capacity. The mechanism of the inhibitory effect of the JQ1 and ingenol-B combination relative to ingenol-B alone was unclear but the effect appeared to be dose dependent.

**Conclusions:**

Ingenol-B does not inhibit HIV-specific CD8+ T cell responses in vitro. These responses are however modestly inhibited when 100 nMingenol-B is combined with JQ1. Since HIV-specific CD8+ T cell activity may be essential for the eradication of reactivated latently infected cells, the potency of latency-reversal activity of drug combinations must be balanced against the effects of the combinations on HIV-specific CD8+ T cell responses.

## Introduction

Shock and kill strategies have been proposed as a possible mechanism for HIV-1 eradication [1, 2]. The strategies involve the use of latency-reversing agents (LRAs) such as Histone Deacetylase (HDAC) inhibitors and PKC-agonists to “shock” latently infected CD4+ T cells and myeloid cells into producing viral proteins that could then be recognized by effector cells leading to the “kill” component of the strategy. Several studies have shown that LRAs do in fact lead to an increase in HIV transcription and/or blips of viremia in clinical trials; however this has not been accompanied by a decrease in the size of the latent reservoir [3, 4, 5, 6]. One possible reason for this disconnect is that the number of latently infected cells that were eradicated in these studies represent a very small proportion of the total latent reservoir. Another potential explanation is that the LRAs inhibit the responses of HIV-specific effector cells thereby leading to ‘shocking’ without ‘killing’.

Recent studies have indeed shown that different classes of LRAs inhibit the responses of natural killer (NK) cells [7,8], T cells [9], and HIV-specific CD8+ T cells [10, 11, 12]and others may affect the susceptibility of CD4+ T cells to HIV-1 infection [13]. We previously showed that the PKC-agonist bryostatin-1 inhibited the suppressive capacity of primary CD8+ T cells from elite controllers/suppressors (ES) whereas the PKC-agonist prostratin had no effect on these cells [11]. Furthermore, drug combinations that were shown to have synergistic effects on latency reversal [14] also had different effects on HIV-specific CD8+ T cells [11]. For instance, the combination of bryostatin-1 with the HDAC inhibitor romidepsin induced more inhibition of the suppressive capacityof T cells than either drug alone, whereas the combination of prostratin with the bromodomaininhibitor JQ1 did not significantly impact the HIV-specific response [11].

Ingenol-B is a relatively new addition to the gradually increasing list of potential candidates for latency reversal [15, 16]. It is derived via a series of chemical reactions that result in a selective esterification at the carbon 3 position of ingenol obtained from ingenol esters of the *Euphorbiaceatirucalli* plant [17]. While ingenol itself is thought to be less efficient at enhancing viral replication [18], its derivatives, such asingenol-B andingenoldibenzoate, are known to demonstrate potent latency reversal when used both alone [19, 20] and in combination with JQ1 in vitro [15,21], and also when used with the HDAC inhibitor vorinostat in vivo in a recent SIV study [22]. This synergy of viral reactivation exhibited when ingenol derivatives are combined with other LRAs likely stems from the different, but complimenting, mechanisms through which each drug reactivates viral LTR- with ingenol achieving this via the PKC-NFκB pathway, and JQ1 and vorinostat doing so via p-TEFbrecruitment, and HDAC inhibition, respectively.

Given their efficacy at latency reversal both alone and in combination with other drugs such as JQ1, ingenol derivatives appear to be promising candidates for the shockandkill cure agenda. However, while much has been done to assess its potency as an LRA, no studies have assessed the effect of the drug on the ability of HIV-specific CD8+ T cells to directly inhibit viral replication. This question is addressed in this study with a focus on the effect of the ingenol derivative ingenol-B on CD8+ T cells when used either alone or in combination with the bromodomain inhibitor JQ1.We also assessed the effects of other PKC agonists, and their combinations, on CD8+ T cell function, using CD8+ T cells form ES, since the cells of these patients have been shown to have superior, HIV specific suppressive capacities [23, 24, 25, 26, 27, 28, 29, 30]. The results have implications for HIV-1 eradication strategies.

## Materials and methods

### Patients

HIV-1 positive and HIV-1 negative blood samples were obtained from donors with written, informed consent and handled according to a Johns Hopkins University IRB approved protocol. Elite suppressors are patients who have maintained undetectable viral loads without antiretroviral therapy. Chronic progressors (CPs) are patients on antiretroviral therapy who have maintained undetectable viral loads for more than 1 year. Healthy donors (HDs) are HIV negative donors. The clinical characteristics of the ES subjects are summarized in Table 1.

**Table 1:** Clinical characteristics of ESused in the study.

	CD4 count	Race/Gender/Age	First positive HIV test	HLA-A	HLA-B
ES3	1149	AA/F/65	1991	25,68	51,57
ES5	617	AA/F/65	1990	23,68	57,58
ES6	601	AA/F/60	1992	23	15,57
ES9	711	AA/F/66	1999	2,30	27,57
ES22	1141	AA/M/55	2009	30,31	15,57
ES24	1452	AA/M/63	2009	24,30	7,57
ES31	1326	AA/F/63	2008	3	27,58
ES48	978	H/F/33	2012	3,23	44,51

### The effect of LRAs on elite suppressor CD8+ T cell suppressive capacity

**Fig 1:**
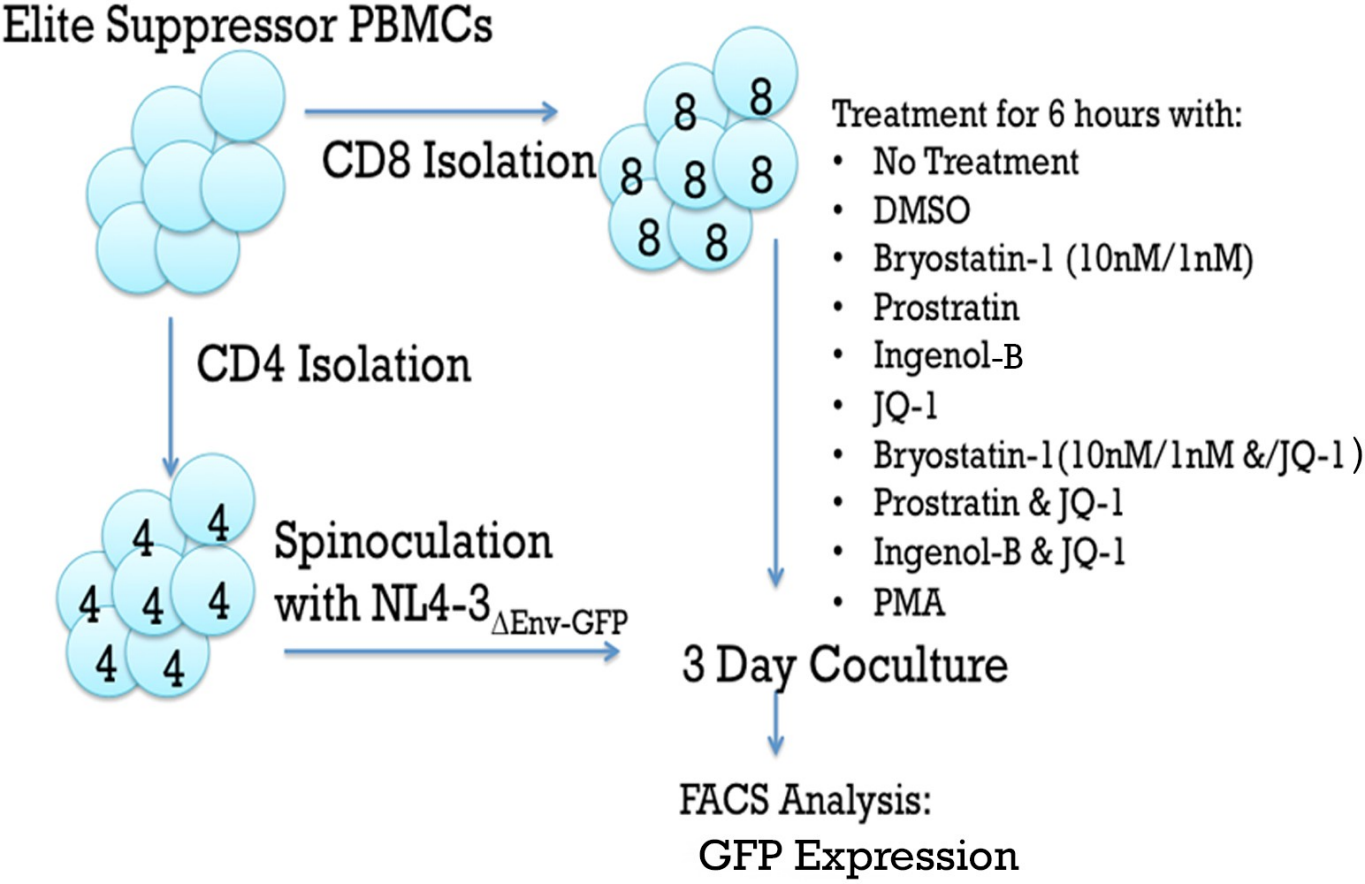
A representative flow diagram of the suppression assay.

Peripheral blood mononuclear cells (PBMCs) were obtained from whole blood via Ficoll-Paque PLUS gradient centrifugation (GE Healthcare Life Sciences).CD8 + T cells were isolated from PBMCs of elite suppressors by positive selection with Miltenyi beads. CD4+ T cells were isolated from the CD8+ T cell negative cells by negative selection using Miltenyi beads. CD8 + T cells from elite suppressor patients were treated for six hours with either media, DMSO (negative control, 0.1%), JQ1 (1 μM; Sigma Aldrich), ingenol-B (100nM, Amazônia Fitomedicamentos Ltda.), bryostatin-1 at two concentrations (10 nM, 1 nM), prostratin(0.3 μM; Sigma Aldrich), or PMA (positive control, 50ng/mL, Sigma). In some experiments, the PKC-agonists were combined with JQ1 at the same concentrations. All drug concentrations were selected based on either physiological relevance in clinical studies (bryostatin at 1nM) or previous work that demonstrated them to be optimal for latency reversal [14, 31]. Bulk CD4 + T cells were spinoculated at 1200 × g for two hours at 37°C with HIV-1NL4 − 3 ∆ Env − GFP at 200ng/100,000 cells. HIV-1NL4 − 3 ∆ Env − GFP is a replication incompetent lab strain pseudovirus with *env* replaced with *gfp* and whose expression is controlled by the HIV promoter. At the conclusion of the six-hour drug treatments, the CD8 + T cells were washed and addedat a 1:1 effector:target ratio to the spinoculated CD4 + T cells. The cells were co-cultured for three days prior to Flow cytometry (FACS) analysis (Fig 1).

### Effects of latency reversing agents on cell death, proliferation, exhaustion, and activation immune markers

PBMCs were isolated from HDs or CPs, seeded at 1x10^6^/well in a 48 well plate and treated for 6 hours at 37^°^C with the individual LRAs or LRA combinations listed above. Cells were washed three times after drug treatment and re-seeded into 96 well plates at 200,000 cells/well/treatment type and assessed for immune marker expression via FACS at four time points: 6 hours (immediately after drug treatment), 24 hours, 48 hours and 72 hours. We also explored the potential of select LRAs/LRA combinations, to either induce proliferation, or increase cell death, in CD8+ T cells isolated from healthy donor PBMCs. In this assay, CD4+ and CD8+ T cells were isolated from donor PBMCs and combined at a 1:1 ratio. They were washed with pre-warmed PBS and exposed to 1uM CFSE (diluted in DMSO to 5uM, then warm PBS to 1uM) at a 1x10^6^cells/ml of CFSE with gentle vortexing. Cells were then incubated for 10mins at 37^°^C and CFSE quenched with 5 times original volume of cold media followed by a5 minute incubation at 4^°^C. Cells were washed one more time with media and treated for 6 hours with select LRAs or LRA combinations in 96 well plates at 200,000 cells/well/treatment. Drug combinations are the same as used a prior, except for the addition of ingenol-B at 10nM and PMA/Iono at 50ng/ml and 1uM respectively. At the end of this incubation, cells were extensively washed, and cultured for an additional 3 days, after which cell death and proliferation were assessed by FACS. For Ki-67 staining, cells were treated with drugs for 6 hours prior to cell culture. On day 3, antibodies to surface markers were added and the cells were then fixed with 70% cold ethanolfollowed by intracellular staining with the Ki-67 antibody (Biolegend).

### FACS analysis

For the suppression assay, the following antibodies were used: CD3 (Pacific Blue, clone UCHT1, BioLegend), CD8 (PE, clone RPA-T8,BDPharmingen) and CD69 (APC, clone FN50, BioLegend). The percent infection was assessed by quantifying GFP expression using the following formula:

[1−(%GFP+CD4+TcellsculturedwithCD8+Tcells)/(%GFP+CD4+Tcellswithouteffectors)x100%].

The following markers were used to assess cell death, immune activation, and exhaustion: CD3 (PE, clone UCHT1, BD Pharmingen), CD8 (APC-H7, clone SK1, BD Biosciences), CD69 (APC, clone FN50, BioLegend), 7AAD (Read in PerCP/Cy5.5, BD Pharmingen), Annexin V (Read in V450, BD Biosciences) and PD1 (PerCP/Cy5.5, clone EH12.2H7, BioLegend)CD25 (FITC, clone M-A251, BD Pharmingen), HLA-DR (PE, clone L243, Biolegend) and Ki67 (APC, clone Ki67, Biolegend). Proliferation was assessed using the CellTrace cell proliferation kit from Invitrogen, and read in the FITC channel.

### Statistics

All statistical analysis and associated graphs were generated in GraphPad Prism 7. For each parameter studied, statistical significance was assessed using a one-way ANOVA with repeated measures. The Geisser-Greenhouse Correction was also employed to account for violations of sphericity. In experiments that entailed replicates, standard deviations were used to determine variances from corresponding means. In all cases, the Dunnett’s Multiple Comparison’s test was used to assess variations of treatment groups from the DMSO control. Multiplicity adjusted P values were reported to assess significance with the following p-value demarcations: ns (p>0.05), * (p<0.05), ** (p<0.01), *** (p<0.001), **** (p<0.0001).

## Results

### Ingenol-B does not inhibit ES CD8+ T cell suppressive capacity

**Fig 2:**
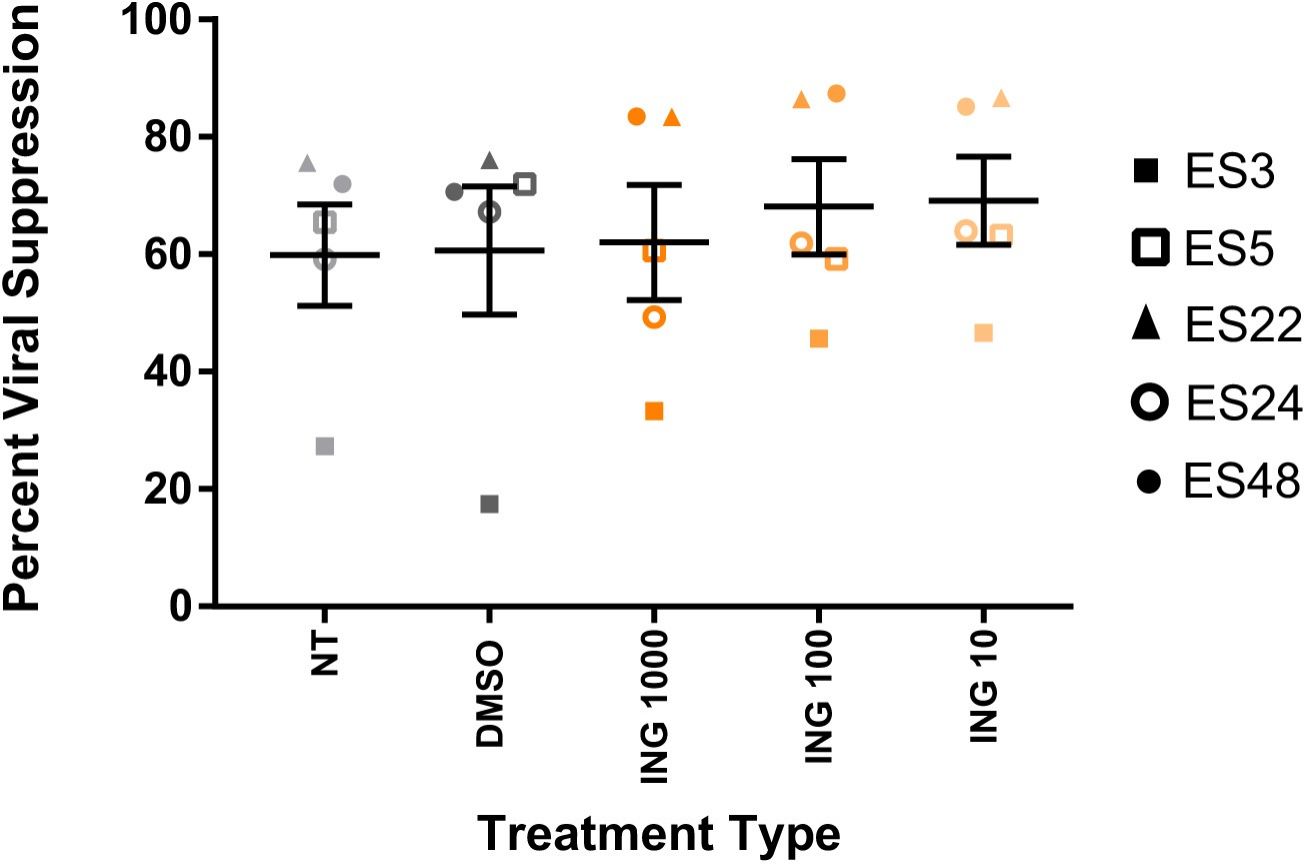
Elite suppressor CD8 + T cell responses are not inhibited by ingenol-B. CD8 + T cells from 5ES were either not treated (NT) or pre-incubated with DMSO oringenol-B at 3 different doses for six hours, washed, then added to autologous CD4 + T cells infected with a lab strain HIV-1 pseudovirus at a 1:1 effector:target ratio. Percent suppression of viral replication was determined after 3 days. Triplicates were performed and mean values are shown for each individual.

To determine the effect of individual drugs on the suppressive capacity of HIV-specific CD8+ T cells, we treated CD8+ T cells isolated from elite suppressors for 6hrs with three different doses of ingenol-B that have been tested in vitro, and then co-cultured them (after extensive washes) over a 3-day period with autologous CD4+ T cells spinoculated with a lab strain HIV pseudovirus. There was no inhibition of CD8+ T cell suppressive capacity with 10 nM, 100nM or 1000nM of the drug compared to the DMSO control (Fig 2). We therefore selected the dose of 100nM for comparisons to other LRAs including the bromodomain inhibitor JQ1 and the PKC agonists bryostatin-1 and prostratin.

### The combination of ingenol-B and JQ1 has a modest inhibitory effect on ES CD8+ T cell suppressive capacity

**Fig 3:**
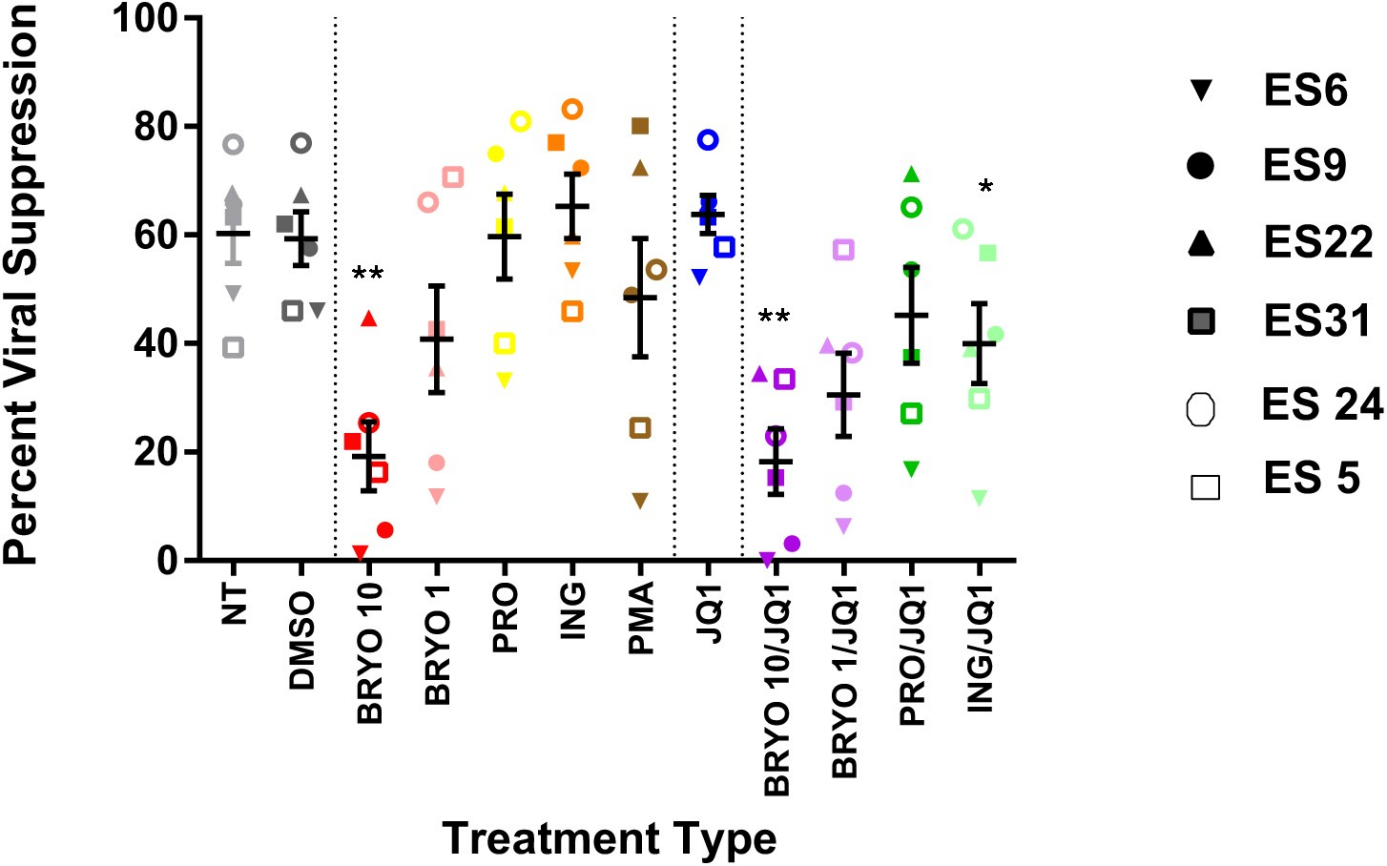
Elite suppressor CD8 + T cell responses are modestly inhibited by the combination of ingenol-B and JQ1. CD8 + T cells from 6 ES were pre-incubated with indicated LRAs for six hours, washed, then added to autologous CD4 + T cells infected with a lab strain HIV-1 pseudovirus at a 1:1 effector:target ratio. Percent suppression of viral replication was determined after 3 days. Triplicates were performed and mean values are shown for each individual. One-way repeated measures ANOVAs were used to determine significance for each of the two sets of experiments. Symbols directly above treatments indicate differences from the DMSO control. * p< 0.05, ** p < 0.01, *** p < 0.001, **** p < 0.0001. Cells were either non treated (NT) or treated with DMSO (0.1%), bryostatin-1 at 10 or 1 nM (BRYO 10 and BRYO 1 respectively), prostratin at 0.3 uM (PRO), ingenol-B at 100nM (ING), PMA at 50ng/mL (PMA) and JQ1 at 1uM (JQ1). In some experiments, the PKC-agonists were combined with JQ1 at the same concentrations (BRYO 10/JQ1, BRYO 1/JQ1, PRO/JQ1, ING/JQ1).

As previously described [11], bryostatin-1 caused significant inhibition of the suppressive capacity of ES CD8+ T cells at 10nM (19.22% suppression versus 59.33% for DMSO treated cells, p = 0.002) whereas prostratin and JQ1 individually had no effect (59.74% suppression and 63.77% suppression respectively, Fig 3). CD8+ T cells treated with 100nM ingenol-B for 6 hours suppressed viral replication as efficiently as CD8+ T cells treated with the DMSO vehicle (65.33% suppression, p = 0.567, Fig 3).While neither100 nM of ingenol-B nor JQ1 had a significant effect on ES CD8+ T cells, the combination of the 2 drugs had a modest, but significant, inhibitory effect on the suppressive capacity of the T cells (39.99% suppression compared to 59.33% suppression with DMSO, p = 0.033). A more pronounced inhibitory effect was seen with the combination of bryostatin-1 (10nM) and JQ1 (18.23% suppression), and no significant inhibition was seen with the prostratin/JQ1 combination (45.23% suppression) as previously described [11].

### Ingenol-B induces high levels of CD8+ T cell activation, modest cell death and PD1 expression and no cell proliferation

**Fig 4:**
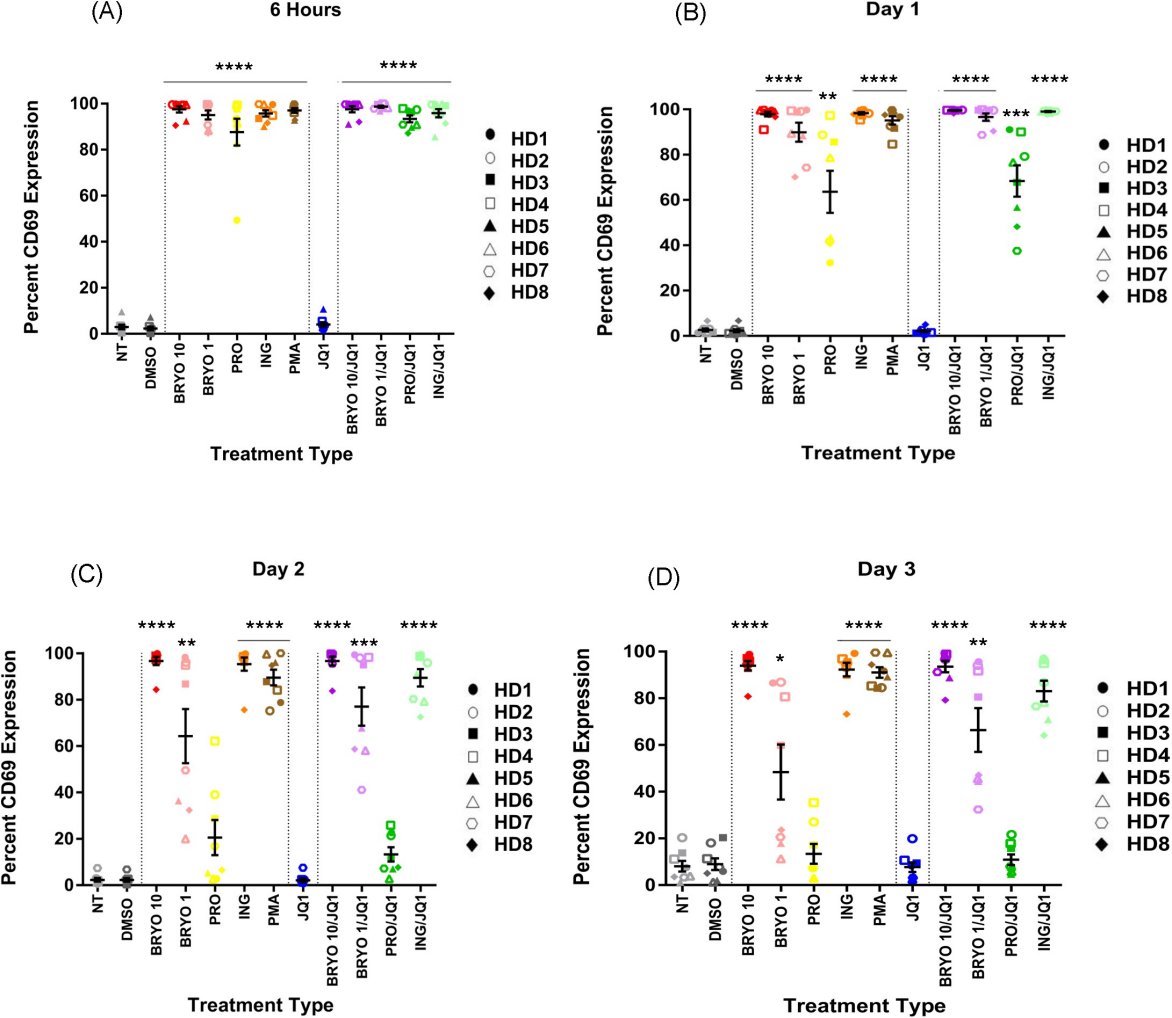
CD69 expression is upregulated following treatment with PKC-agonists. PBMCs from 8 HDs were treated with LRAs for 6 h, washed, then cultured for up to three days and examined for CD69 expression on CD8 + T cells. Mean expression ± standard error is indicated for each treatment at (A) 6 hours, (B) day 1, (C) day 2 and (D) day 3. Symbols directly above treatments indicate differences from the DMSO control. * p< 0.05, ** p < 0.01, *** p < 0.001, **** p < 0.0001. Cells were either non treated (NT) or treated with DMSO (0.1%), bryostatin-1 at 10 or 1 nM (BRYO 10 and BRYO 1 respectively), prostratin at 0.3 uM (PRO), ingenol-B at 100nM (ING), PMA at 50ng/mL (PMA) and JQ1 at 1uM (JQ1). In some experiments the PKC-agonists were combined with JQ1 at the same concentrations (BRYO 10/JQ1, BRYO 1/JQ1, PRO/JQ1, ING/JQ1).

In order to address the different effects of the inhibitory (bryostatin-1) and non-inhibitory (prostratin and ingenol-B) PKC-agonists, we measured the amount of immune activation they induced on CD8+ T cells from healthy donors (Fig 4). With the exception of JQ1, which slightly increased levels of CD69, all drugs induced high levels of CD69 expression after 6 hours of drug exposure. This activation was sustained for bryostatin-1 and ingenol-B, but by the 24 hour time point, CD69 expression on CD8+ T cells exposed to prostratin had begun to decline and by 2days there was no difference in the level of expression between prostratin and DMSO treated cells. Similarly, besides prostratin and bryostatin at 1nM, cells treated with the combination of JQ1 and PKC agonists had sustained levels of activation as cells treated with the PKC-agonists alone. A similar pattern was seen with cells obtained from chronic progressors on ART (S1 Fig). We also examined other activation markers and found that aside from PMA, PMA/Iono, none of the LRAs induced significant expression of CD25 (S2 Fig) or co-expression of HLA-DR and CD38 (S3 Fig).

**Fig 5:**
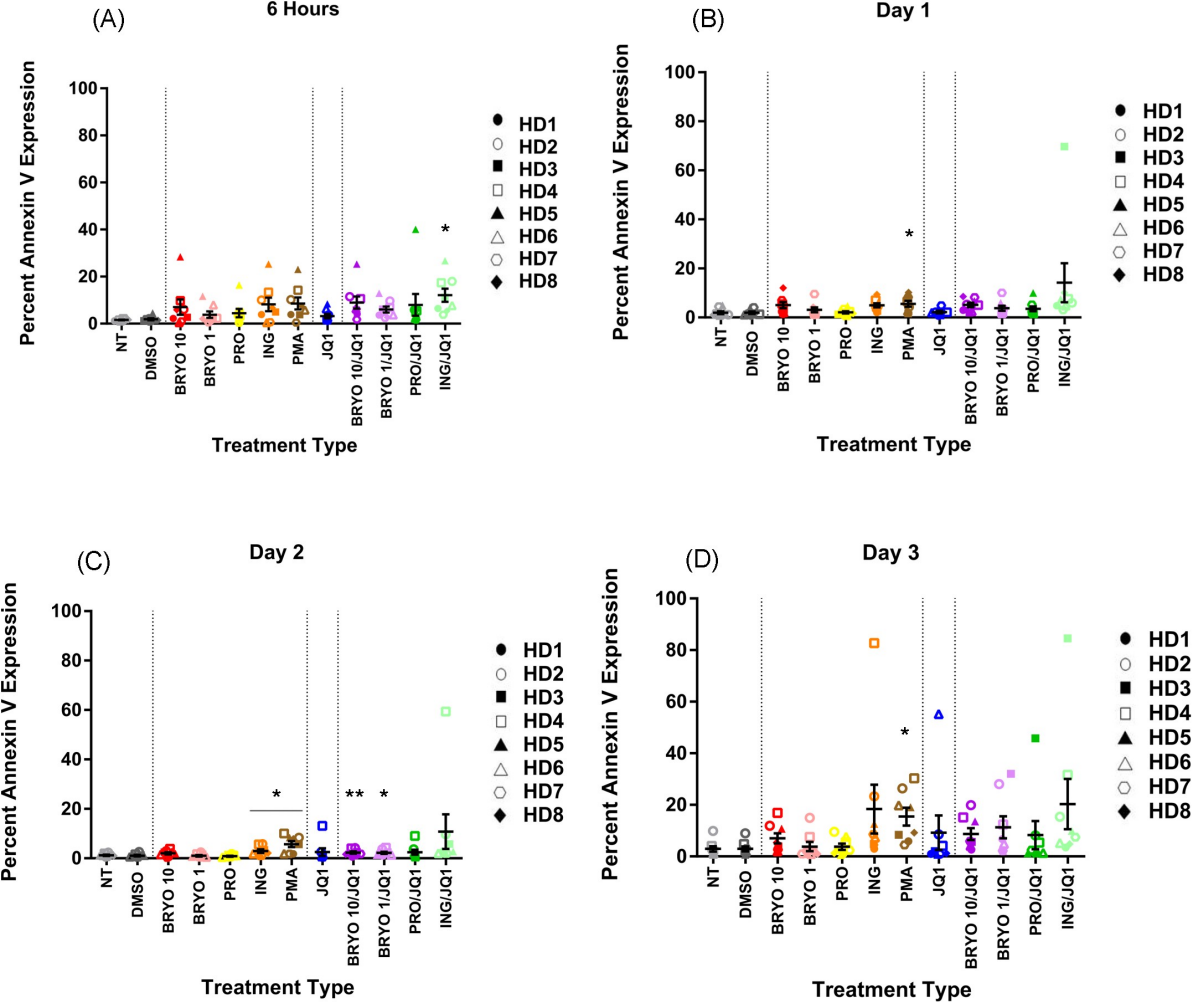
Ingenol-B and ingenol-B/JQ1 combination causes modest levels of cell death in CD8+ T cells. PBMCs from eight HDs were isolated and treated with LRAs for 6 h, washed, then cultured for up to three days and examined for cell death as measured by percent annexin V expression. Mean expression ± standard error is indicated for each treatment at (A) 6 hours, (B) day 1, (C) day 2 and (D) day 3. Symbols directly above treatments indicate differences from the DMSO control. * p< 0.05, ** p < 0.01, *** p < 0.001, **** p < 0.0001. Cells were either non treated (NT) or treated with DMSO (0.1%), bryostatin-1 at 10 or 1 nM (BRYO 10 and BRYO 1 respectively), prostratin at 0.3 uM (PRO), ingenol-B at 100nM (ING), PMA at 50ng/mL (PMA) and JQ1 at 1uM (JQ1). In some experiments the PKC-agonists were combined with JQ1 at the same concentrations (BRYO 10/JQ1, BRYO 1/JQ1, PRO/JQ1, ING/JQ1).

Ingenol-B treatment resulted in a modest but significant increase in annexin V expression at 2 days, but no significant increase in cell death was observed at any other time point (Fig 5 and S4 Fig).The percentages of CD8+ T cells expressing PD1 after ingenol-B and ingenol-B/JQ1 exposures were also slightly elevated at the 1 and 2 day time points but not enough to explain the differences in suppressive capacity ofES CD8+ T cells (S5 Fig and S6 Fig).We also co-cultured purified CD4+ and CD8+ T cells to more closely approximate the experiment we performed with the ES CD8+ T cells and observed no significant increase in CD8+ T cell death on day 3 in this system, following a 6 hour exposure to ingenol-B or ingenol-B and JQ1 (S7 Fig).Additionally, we asked whether non-specific proliferation of CD8+ T cells could explain the difference in responses to ingenol-B versus ingenol-B and JQ1. We found that the LRAs did not induce any non-specific proliferation of CD8+ T cells as determined by CFSE dilution or Ki-67 expression (S8 Fig).

### Ingenol-B and ingenol-B/JQ1 induce transient downregulation of CD3 but have no effect on surface CD8 expression

**Fig 6:**
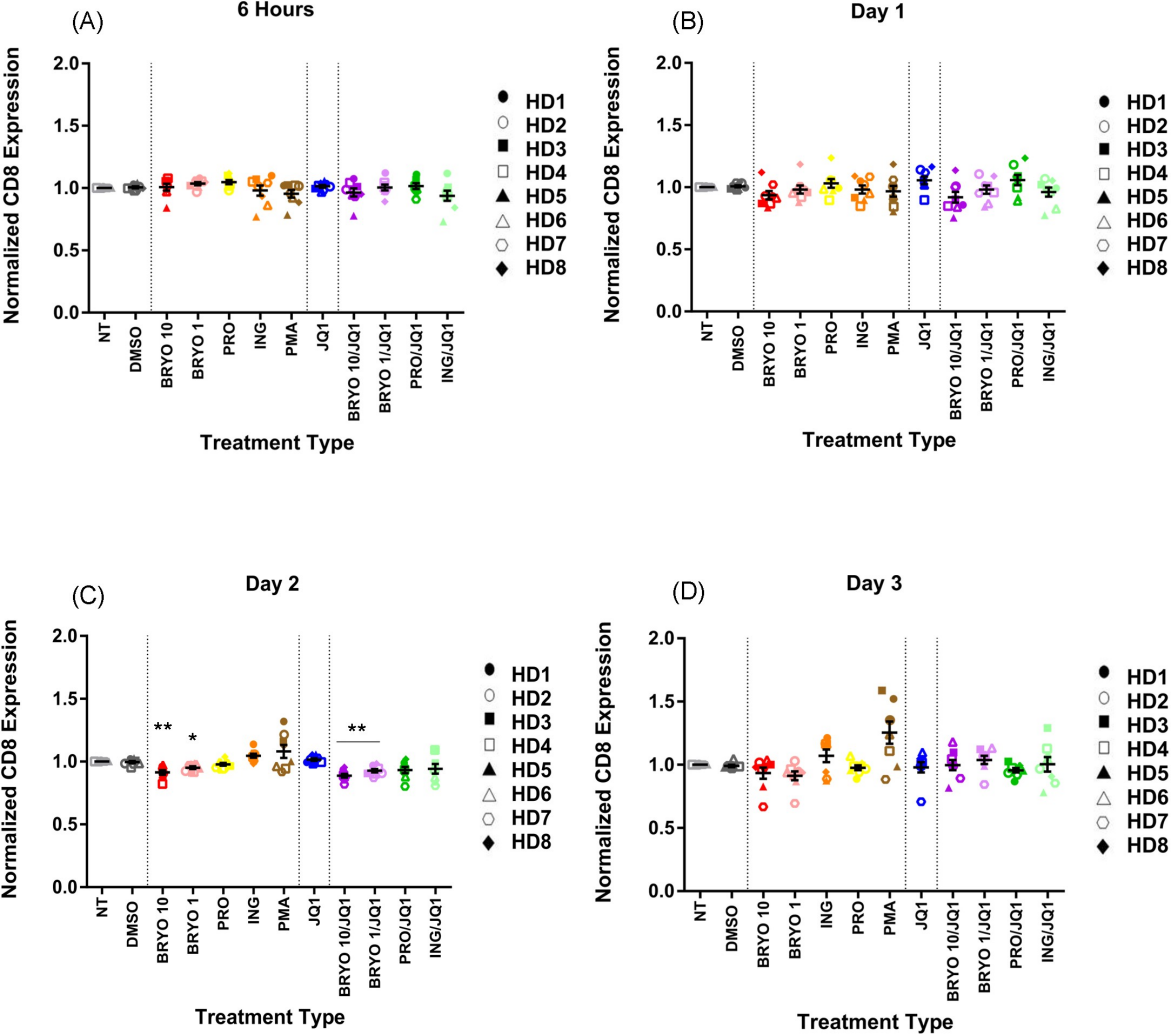
Surface CD8 expression is modestly downregulated by some LRAs. PBMCs from 8 HDs were treated with LRAs for 6 h before being washed and then cultured for up to three days and examined for CD8 expression. Mean expression ± standard error is indicated for each treatment at (A) 6 hours, (B) day 1, (C) day 2 and (D) day 3. Symbols directly above treatments indicate differences from the DMSO control. * p< 0.05, ** p < 0.01, *** p < 0.001, **** p < 0.0001. Cells were either non treated (NT) or treated with DMSO (0.1%), bryostatin-1 at 10 or 1 nM (BRYO 10 and BRYO 1 respectively), prostratin at 0.3 uM (PRO), ingenol-B at 100nM (ING), PMA at 50ng/mL (PMA) and JQ1 at 1uM (JQ1). In some experiments the PKC-agonists were combined with JQ1 at the same concentrations (BRYO 10/JQ1, BRYO 1/JQ1, PRO/JQ1, ING/JQ1).

**Fig 7:**
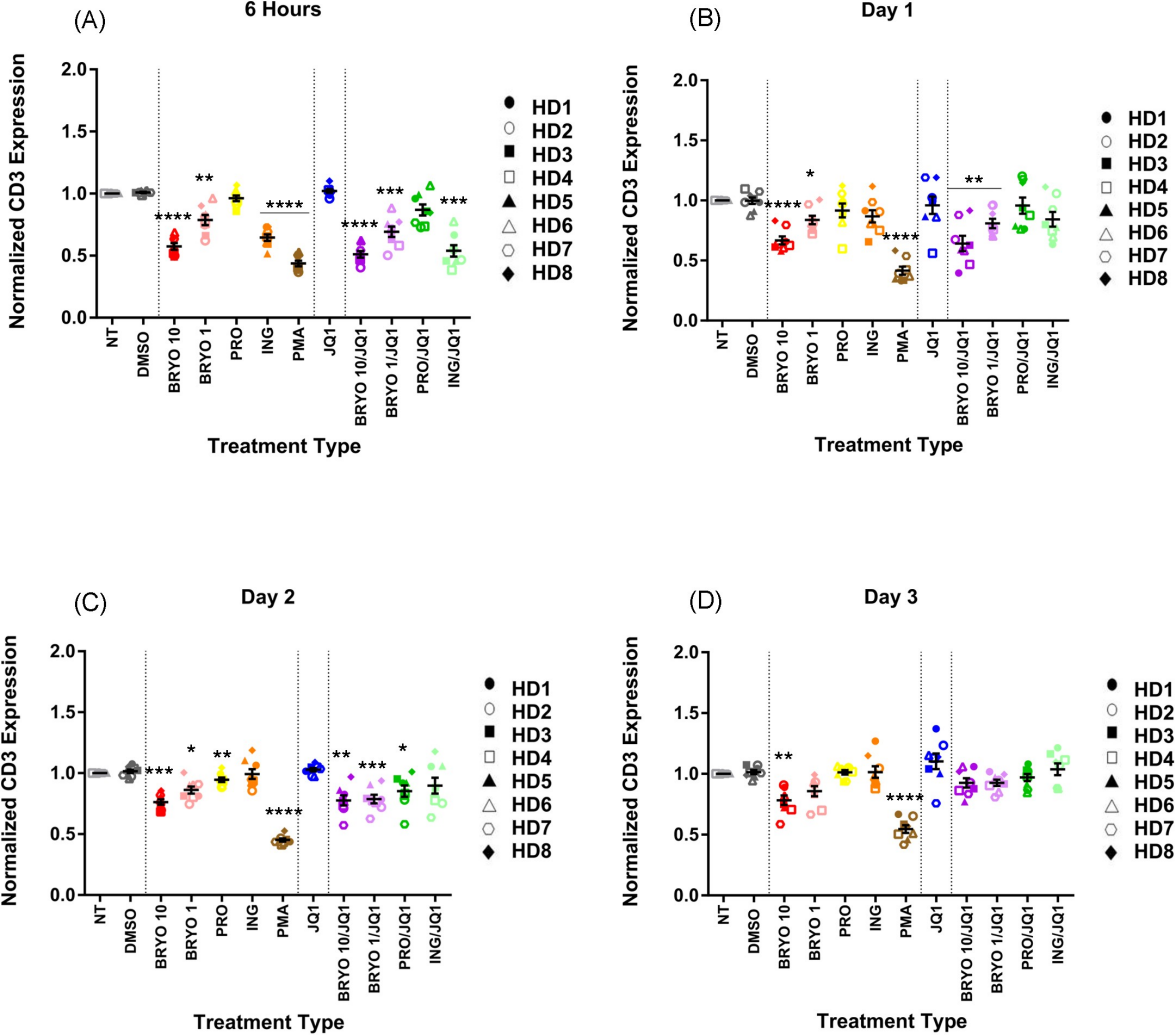
Surface CD3 expression is downregulated following treatment with PKC-agonists. PBMCs from 8 HDs were treated with LRAs for 6 h before being washed and then cultured for up to three days and examined for CD3 expression. Mean expression ± standard error is indicated for each treatment at (A) 6 hours, (B) day 1, (C) day 2 and (D) day 3. Symbols directly above treatments indicate differences from the DMSO control. * p< 0.05, ** p < 0.01, *** p < 0.001, **** p < 0.0001. Cells were either non treated (NT) or treated with DMSO (0.1%), bryostatin-1 at 10 or 1 nM (BRYO 10 and BRYO 1 respectively), prostratin at 0.3 uM (PRO), ingenol-B at 100nM (ING), PMA at 50ng/mL (PMA) and JQ1 at 1uM (JQ1). In some experiments the PKC-agonists were combined with JQ1 at the same concentrations (BRYO 10/JQ1, BRYO 1/JQ1, PRO/JQ1, ING/JQ1).

Prior studies have shown that ingenol-B causes a significant downregulation of surface CD4 expression [19, 20] and thus we asked whether a similar downregulation of CD8 might play a role in the inhibitory effect of the ingenol-B/JQ1 combination. We found that while CD8 surface levels were slightly lower in cells treated with bryostatin-1at the day 2 time point (normalized MFI of 0.914compared to DMSO treated cells of 0.996, p = 0.009), cells treated with 100nM ingenol-B and ingenol-B/JQ1 had similar levels of CD8 as cells treated with the DMSO vehicle (normalized MFIs of 1.046 and 0.943 respectively, Fig 6). In contrast, CD3 was significantly downregulated on CD8+ T cells that were treated with bryostatin-1at 10nM and 1nM (normalized MFIs of 0.574 and 0.787 respectively), ingenol-Bat 100nM (normalized MFI of 0.645),bryostatin/JQ1 (normalized MFI of 0.5113) and ingenol-B/JQ1(normalized MFI of 0.539)at the 6 hour time point (Fig 7 and S9 Fig). Interestingly, this downregulation was transient for ingenol-B and ingenol-B/JQ1 treated cells, such that CD3 had normalized by the 24-hour time point. In contrast, cells treated with bryostatin-1 and bryostatin-1/JQ1 had sustained downregulation of CD3 (Fig 7).

### The combination of a lower dose of Ingenol-B with JQ1 has no significant inhibitory effect on ES CD8+ T cell suppressive capacity

**Fig 8:**
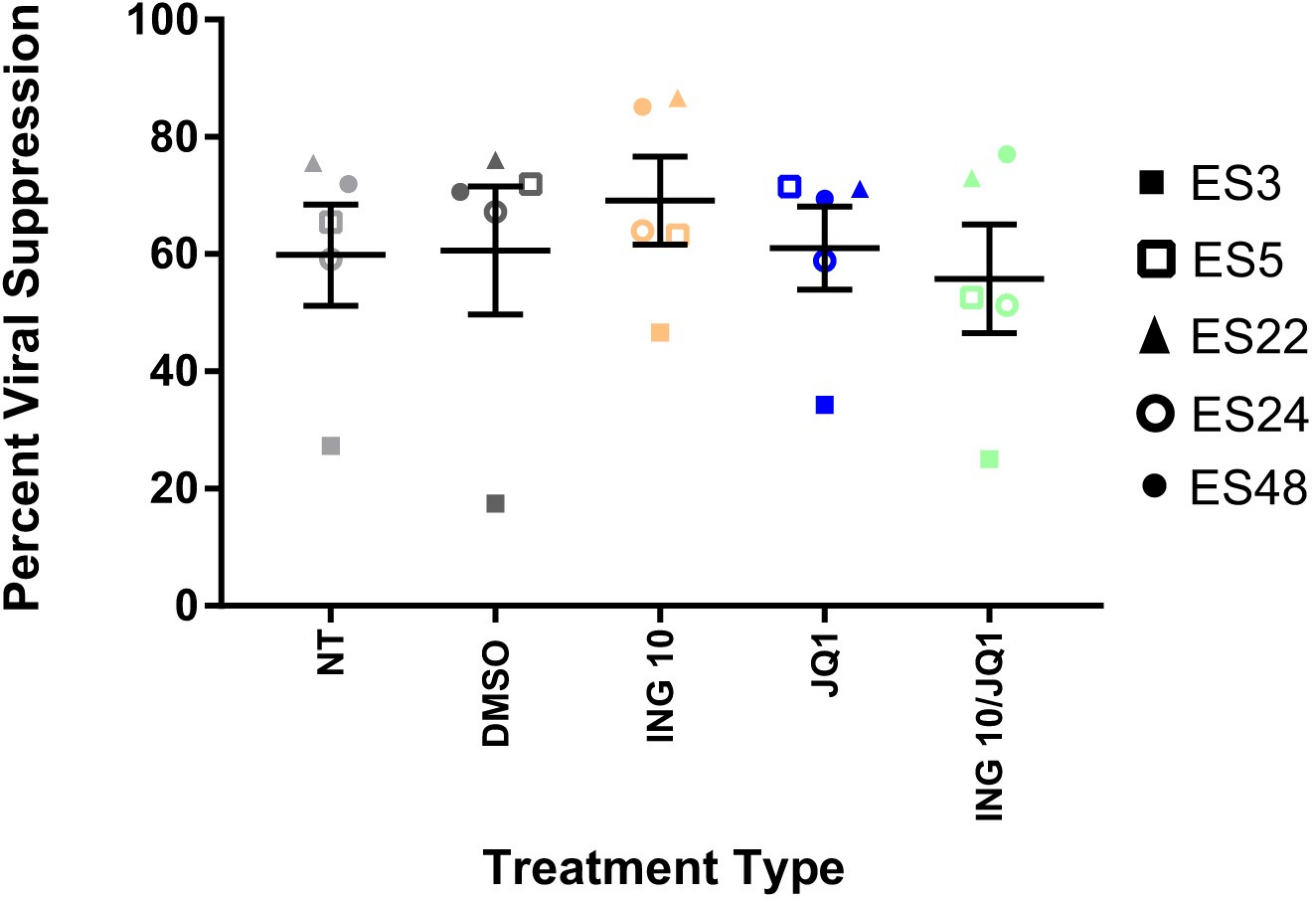
Elite suppressor CD8 + T cell responses are not inhibited by 10nMingenol-B in combination with JQ1. CD8 + T cells from 5ES were pre-incubated with 10nMingenol-B and 1uM JQ1 for six hours, washed, then added to autologous CD4 + T cells infected with a lab strain HIV-1 pseudovirus at a 1:1 effector:target ratio. Percent suppression of viral replication was determined after 3 days. Triplicates were performed and mean values are shown for each individual.

In order to determine whether the modest inhibitory effect of the combination of ingenol-B with JQ1 was dependent on the dose of ingenol-B, we performed experiments with 10nM of the drug in combination with the same dose of JQ1. As shown in Fig 8, no inhibition of the suppressive capacity of CD8+ T cells was observed with this specific combination.

## Discussion

Ingenol derivatives have been shown to function efficiently both as monotherapy and in combination with other drugs such as JQ1 and vorinostatin vitro [15, 19, 20, 21] and in vivo [22].

While some ingenol derivatives are thought to function via alternative pathways [18], it is generally established that most ingenol derivatives, including ingenol-B, function as PKC agonists, reversing latency via the PKC-NFκB pathway [19, 20, 21].Besides PKC activation, ingenol-B has also been shown to facilitate transcript elongation via the recruitment of specific P-TEFb components namely CDK9 and CyclinT1 [17]. Ingenol-B is also thought to be capable of inhibiting viral propagation by downregulatingHIV receptors and co-receptors (CD4, CCR5 and CXCR4) [19, 20]. This drug may therefore be a promising agent to test in clinical trials.

A recent study has shown that the reversal of latency by itself is not sufficient to eliminate latently infected CD4+ T cells, and that HIV-specific CD8+ T cells may also be needed for the eradication of these cells [32]. This finding has prompted additional studies which have now produced significant data to suggest that some LRAs may inhibit NK cell and CD8+ T cell activity [7, 8, 9, 10, 11, 12] and thus may not be the optimal candidates for shock and kill strategies. In a recent study, we showed that agents such as the HDAC inhibitors romidepsin and panobinostat, and the PKC agonist bryostatin-1, inhibited the responses of ES CD8+ T cells in viral suppression assays [11]. We also tested combinations of drugs that had been found to be synergistic and found that some combinations like romidepsin and bryostatin-1, inhibited CD8+ T cell responses to a greater degree than either drug alone.

In this study, we screened ingenol-B to determine whether it inhibited HIV-specific CD8+ T cell responses. We used CD8+ T cells from ES since these patients have potent HIV-specific CD8+ T cell responses that are probably responsible for their control of HIV-1 infection [23, 24, 25, 26, 27, 28, 29, 30]. While HIV-1 specific CD8+ T cells in these subjects are not representative of the CD8+ T cell responses seen in the vast majority of HIV-infected subjects, it is possible that successful therapeutic vaccination of these subjects will enhance CP HIV-specific CD8+ T cell responses to a similar level. We found that neither ingenol-Bat 100 nM nor JQ1 individually inhibited the suppressive capacity of ES CD8+ T cells, but the combination of the two drugs had a modest inhibitory effect. While a difference in CD3 downregulation kinetics could potentially, partially, explain the difference in the effects of bryostatin-1 and ingenol-B on HIV-specific CD8+ T cells, it does not explain the difference observed between ingenol-B and ingenol-B/JQ1 treated cells.

Besides CD3 modulation, we also looked at the expression levels of other cell surface markers such as CD8, annexin V and PD1, to determine if these could explain the difference in suppressive capacities of CD8+ T cells treated with either ingenol-B or its combination with JQ1. We found that ingenol-B alone and in combination with JQ1 does not significantly modulate CD8 or PD1expression on T cells. We also found that treatment with ingenol-B or ingenol-B/JQ1 resulted in minimal cell death which confirms studies by Abreu et al [19].

Our data are also consistent with a recent study that showed that treatment of peripheral blood mononuclear cells from healthy donors and HIV-infected patients with ingenol-B increased the number of antigen specific CD8+ T cells that expressed perforin and CD107a [12].However, while the drug had no effect on the total number of CD8+ T cells that proliferated in response to antigen over a 5 day period, it inhibited the number of times antigen-specific CD8+ T cells proliferated [12]. Taken together, it is possible that while this drug may not have an effect on the short term ability of HIV-specific CD8+ T cells to control infected CD4+ T cells, it could havea negative impact on the ability of HIV-specific CD8+ T cells to expand in vivo. Clinical trials will be needed to assess the effects of this drug alone, and in combination with JQ1, on HIV-specific CD8+ T cells in vivo. The effects of the drugs will likely be dose dependent and while we used concentrations of LRAs that have reversed latency in vitro, it is not clear that these are concentrations of ingenol-B and JQ1 that can be safely achieved in vivo.

In summary, our data suggest that drug combinations should be tested before being used in clinical trials because even LRAs that do not inhibit HIV-specific CD8+ T cell responses by themselves may have significant effects when they are used in combination with each other. Furthermore, while certain combinations of LRAs may be more potent at inducing viral transcription than individual LRAs, the advantages of this enhanced potency may be neutralized by the inhibitory effects of the drug combinations on HIV-specific CD8+ T cell responses.

## Supporting information

S1 Fig CD69 expression is upregulated following treatment with PKC-agonists. PBMCs from 6 CPs were treated with LRAs for 6 h, washed, then cultured for up to three days and examined for CD69 expression on CD8 + T cells. Mean expression ± standard error is indicated for each treatment at (A) 6 hours, (B) day 1, and (C) day 3. Symbols directly above treatments indicate differences from the DMSO control. * p< 0.05, ** p < 0.01, *** p < 0.001, **** p < 0.0001. Cells were either non treated (NT) or treated with DMSO (0.1%), bryostatin-1 at 10nM (BRYO), ingenol-B at 1000, 100 or 10nM (ING 1000, ING 100, ING 10 respectively), PMA at 50ng/mL (PMA) and JQ1 at 1uM (JQ1). In some experiments the PKC-agonists were combined with JQ1 at the same concentrations (BRYO 10/JQ1, ING 100/JQ1). PMA and ionomycin (PMA/IONO, 50ng/ml and 1uM respectively) were used as a positive control.
(TIF)

S2 Fig CD25 expression is not upregulated following treatment with LRAs. PBMCs from 6 CPs were treated with LRAs for 6 h, washed, then cultured for up to three days and examined for CD25 expression on CD8 + T cells. Mean expression ± standard error is indicated for each treatment at (A) 6 hours, (B) day 1, and (C) day 3. Symbols directly above treatments indicate differences from the DMSO control (* p< 0.05). Cells were either non treated (NT) or treated with DMSO (0.1%), bryostatin-1 at 10nM (BRYO), ingenol-B at 1000, 100 or 10nM (ING 1000, ING 100, ING 10 respectively), PMA at 50ng/mL (PMA) and JQ1 at 1uM (JQ1). In some experiments the PKC-agonists were combined with JQ1 at the same concentrations (BRYO 10/JQ1, ING 100/JQ1). PMA and ionomycin (PMA/IONO, 50ng/ml and 1uM respectively) were used as a positive control.
(TIF)

S3 Fig CD38 and HLA-DR co-expression is not upregulated following treatment with LRAs. PBMCs from 6 CPs were treated with LRAs for 6 h, washed, then cultured for up to three days and examined for CD38 and HLA-DR co-expression on CD8 + T cells. Mean expression ± standard error is indicated for each treatment at (A) 6 hours, (B) day 1, and (C) day 3. Symbols directly above treatments indicate differences from the DMSO control (* p< 0.05). Cells were either non treated (NT) or treated with DMSO (0.1%), bryostatin-1 at 10nM (BRYO), ingenol-B at 1000, 100 or 10nM (ING 1000, ING 100, ING 10 respectively), PMA at 50ng/mL (PMA) and JQ1 at 1uM (JQ1). In some experiments the PKC-agonists were combined with JQ1 at the same concentrations (BRYO 10/JQ1, ING 100/JQ1). PMA and ionomycin (PMA/IONO, 50ng/ml and 1uM respectively) were used as a positive control.
(TIF)

S4 Fig Ingenol-B/JQ1 combination causes modest levels of cell death in CD8+ T cells. PBMCs from 6 CPs were isolated and treated with LRAs for 6 h, washed, then cultured for up to three days and examined for cell death as measured by percent annexin V expression. Mean expression ± standard error is indicated for each treatment at (A) 6 hours, (B) day 1, and (C) day 3. Symbols directly above treatments indicate differences from the DMSO control (* p< 0.05, ** p < 0.01). Cells were either non treated (NT) or treated with DMSO (0.1%), bryostatin-1 at 10 nM (BRYO), ingenol-B at 1000, 100 or 10nM (ING 1000, ING 100, ING 10 respectively), PMA at 50ng/mL (PMA) and JQ1 at 1uM (JQ1). In some experiments the PKC-agonists were combined with JQ1 at the same concentrations (BRYO 10/JQ1, ING 100/JQ1) or with PMA and ionomycin (PMA/IONO, 50ng/ml and 1uM respectively).
(TIF)

S5 Fig Ingenol-B/JQ1 combination causes transient, slightly higher levels of % PD1 expression on HD CD8+ T cells. PBMCs from 8 HDs were treated with LRAs for 6 h before being washed and cultured for up to three days and examined for cell exhaustion as measured by percent PD1 expression. Mean expression ± standard error is indicated for each treatment at (A) 6 hours, (B) day 1, (C) day 2 and (D) day 3. Symbols directly above treatments indicate differences from the DMSO control. * p< 0.05, ** p < 0.01, *** p <0.001, **** p < 0.0001. Cells were either non treated (NT) or treated with DMSO (1%), bryostatin-1 at 10 or 1 nM (BRYO 10 and BRYO 1 respectively), prostratin at 0.3 uM (PRO), ingenol-B at 100nM (ING), PMA at 50ng/mL (PMA) and JQ1 at 1uM (JQ1). In some experiments the PKC agonists were combined with JQ1 at the same concentrations (BRYO 10/JQ1, BRYO 1/JQ1, PRO/JQ1, ING/JQ1).
(TIF)

S6 Fig Ingenol-B/JQ1 combination causes slightly higher levels of % PD1 expression on CP CD8+ T cells. PBMCs from 6 CPs were isolated and treated with LRAs for 6 h, washed, then cultured for up to three days and examined for cell death as measured by percent annexin V expression. Mean expression ± standard error is indicated for each treatment at (A) 6 hours, (B) day 1, and (C) day 3. Symbols directly above treatments indicate differences from the DMSO control (* p< 0.05). Cells were either non treated (NT) or treated with DMSO (0.1%), bryostatin-1 at 10 nM (BRYO), ingenol-B at 1000, 100 or 10nM (ING 1000, ING 100, ING 10 respectively), PMA at 50ng/mL (PMA) and JQ1 at 1uM (JQ1). In some experiments the PKC-agonists were combined with JQ1 at the same concentrations (BRYO 10/JQ1, ING 100/JQ1) or with PMA and ionomycin (PMA/IONO, 50ng/ml and 1uM respectively).
(TIF)

S7 Fig LRAs do not increase levels of cell death in CD8+ T cells following incubation with CD4+ T cells. PBMCs were isolated from 6 healthy donors and CD4+ and CD8+ T cells enriched. Cells were combined at a 1:1 ratio, co-cultured and treated with LRAs for 6hrs after which cells were extensively washed and incubated for an additional 3 days before examining for CD8+ T cell death by percent annexin V expression. Mean expression ± standard error is indicated for each treatment at day 3. No significant differences in cell death were observed, relative to the DMSO control. Cells were either non treated (NT) or treated with DMSO (1%), ingenol-B at 100nM or 10nM (ING 100 or ING 10), PMA at 50ng/mL (PMA), JQ1 at 1uM (JQ1) and ingenol-B/JQ1 combinations at the same concentrations(ING 100/JQ1, ING 10/JQ1).
(TIF)

S8 Fig Ingenol-B does not induce proliferation of CD8+ T cells. (A) PBMCs were isolated from 6 HD PBMCs and CD4+ and CD8+ T cells enriched. Cells were combined at a 1:1 ratio and exposed to CellTrace CFSE. They were treated with LRAs for 6hrs incubated for an additional 3 days before examination for CD8+ T cell proliferation by percent CFSE expression. Mean expression ± standard error is indicated for each treatment at day 3. Cells were either non treated (NT) or treated with DMSO (1%), ingenol-B at 100nM or 10nM (ING 100 or ING 10), PMA at 50ng/mL (PMA), PMA/Ionomycin (PMA/IONO, 50ng/ml and 1uM respectively), JQ1 at 1uM (JQ1) and ingenol-B/JQ1 combinations at the same concentrations(ING 100/JQ1, ING 10/JQ1). (B) PBMCs were isolated from 6 CPs and treated for 6 hours with LRAs. They were then extensively washed and incubated for an additional 3 days before intracellular staining with the Ki-67 monoclonal antibody. Mean expression ± standard error is indicated for each treatment.
(TIF)

S9 Fig Surface CD3 expression is downregulated following treatment with PKC-agonists. PBMCs from 6 CPs were isolated and treated with LRAs for 6 h, washed, then cultured for up to three days and examined for CD3 expression. Mean expression ± standard error is indicated for each treatment at (A) 6 hours, (B) day 1, and (C) day 3. Symbols directly above treatments indicate differences from the DMSO control (* p< 0.05, ** p < 0.01, *** p < 0.001, **** p < 0.0001). Cells were either non treated (NT) or treated with DMSO (0.1%), bryostatin-1 at 10 nM (BRYO), ingenol-B at 1000, 100, or 10nM (ING 1000, ING 100, ING 10 respectively), PMA at 50ng/mL (PMA) and JQ1 at 1uM (JQ1). In some experiments the PKC-agonists were combined with JQ1 at the same concentrations (BRYO 10/JQ1, ING 100/JQ1) or with PMA and ionomycin (PMA/IONO, 50ng/ml and 1uM respectively).
(TIF)

S1 Dataset 6 Hours Data HDs. (XLSX)

S2 Dataset Day1 Data HDs. (XLSX)

S3 Dataset Day2 Data HDs. (XLSX)

S4 Dataset Day3 Data HDs. (XLSX)

S5 Dataset 6Hours Data CPs. (XLSX)

S6 Dataset Day1 Data CPs. (XLSX)

S7 Dataset Day3 Data CPs. (XLSX)

S8 Dataset Day3 Suppression ES. (XLSX)

S9 Dataset Day3 Proliferation HDs/CPs. (XLSX)
