# Supplementary figures and images for: The effect of Ingenol-B on the suppressive capacity of elite suppressor HIV-specific CD8+ T cells
Source: PLoS One. 2017 May 3;12(5):e0174516. doi: 10.1371/journal.pone.0174516 (PMC5414940; doi:10.1371/journal.pone.0174516)

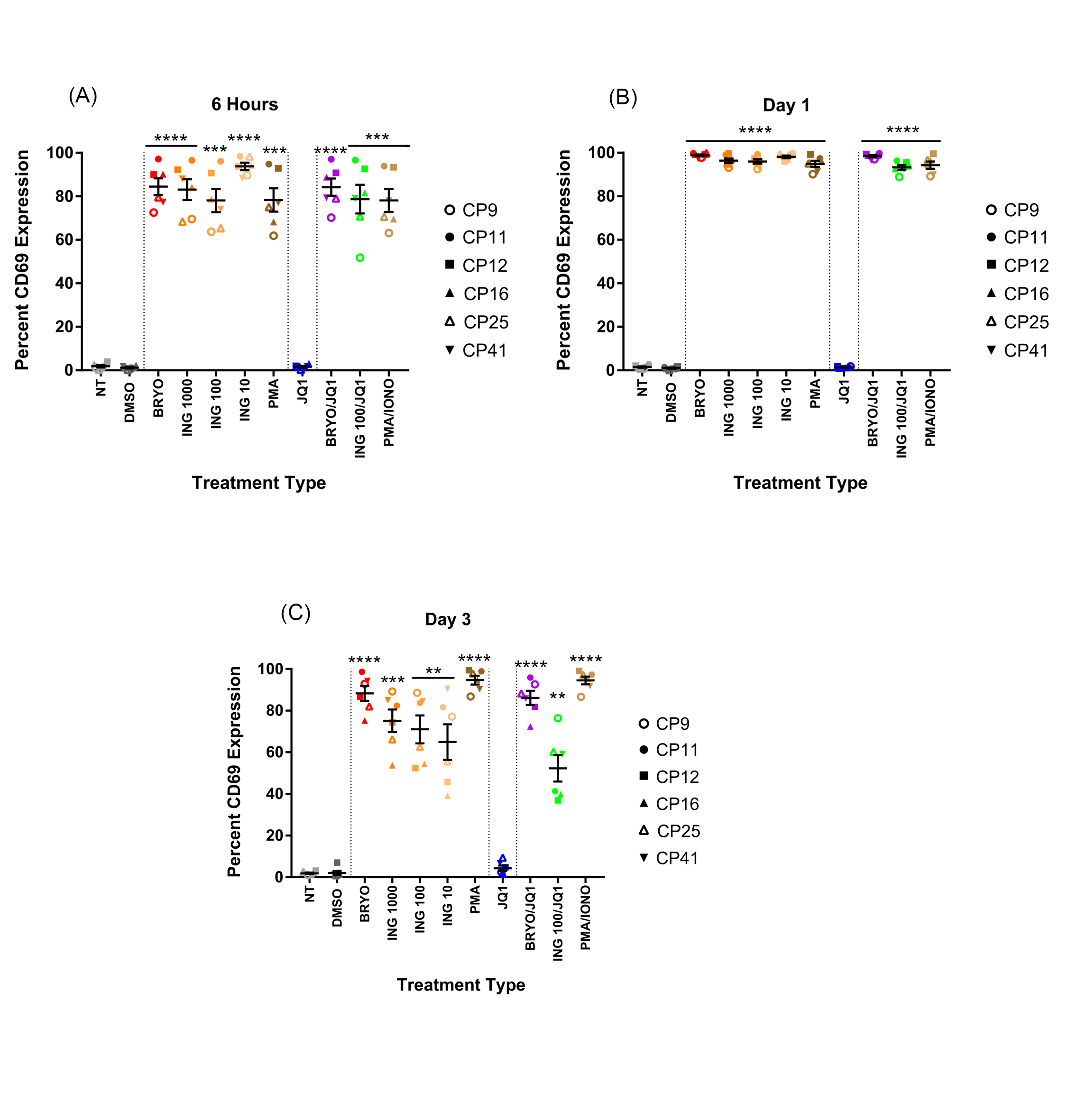

Supplement: S1 Fig — PBMCs from 6 CPs were treated with LRAs for 6 h, washed, then cultured for up to three days and examined for CD69 expression on CD8 + T cells. Mean expression ± standard error is indicated for each treatment at (A) 6 hours, (B) day 1, and (C) day 3. Symbols directly above treatments indicate differences from the DMSO control. * p< 0.05, ** p < 0.01, *** p < 0.001, **** p < 0.0001. Cells were either non treated (NT) or treated with DMSO (0.1%), bryostatin-1 at 10nM (BRYO), ingenol-B at 1000, 100 or 10nM (ING 1000, ING 100, ING 10 respectively), PMA at 50ng/mL (PMA) and JQ1 at 1uM (JQ1). In some experiments the PKC-agonists were combined with JQ1 at the same concentrations (BRYO 10/JQ1, ING 100/JQ1). PMA and ionomycin (PMA/IONO, 50ng/ml and 1uM respectively) were used as a positive control. (TIF) [file pone.0174516.s001.tif]

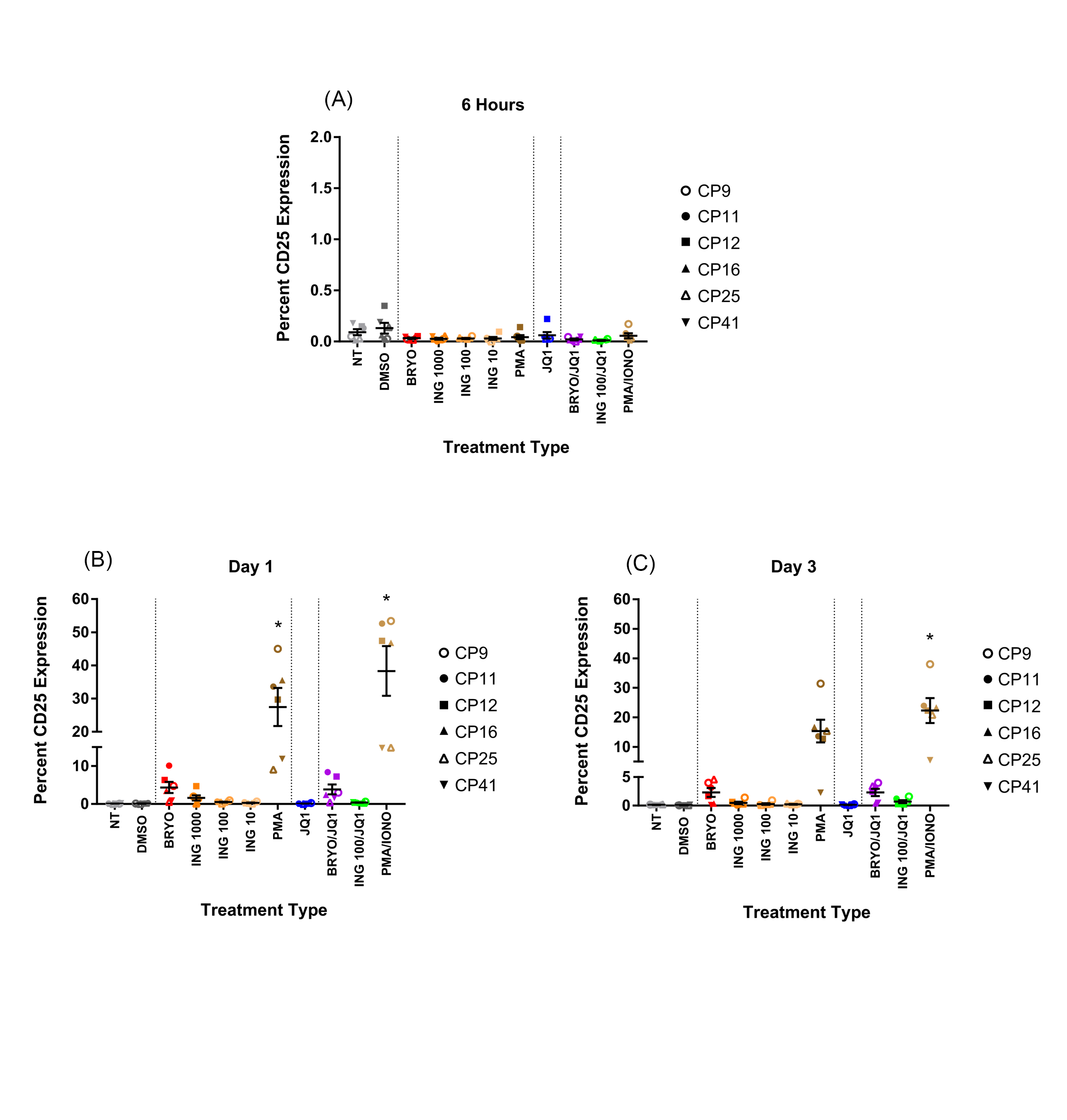

Supplement: S2 Fig — PBMCs from 6 CPs were treated with LRAs for 6 h, washed, then cultured for up to three days and examined for CD25 expression on CD8 + T cells. Mean expression ± standard error is indicated for each treatment at (A) 6 hours, (B) day 1, and (C) day 3. Symbols directly above treatments indicate differences from the DMSO control (* p< 0.05). Cells were either non treated (NT) or treated with DMSO (0.1%), bryostatin-1 at 10nM (BRYO), ingenol-B at 1000, 100 or 10nM (ING 1000, ING 100, ING 10 respectively), PMA at 50ng/mL (PMA) and JQ1 at 1uM (JQ1). In some experiments the PKC-agonists were combined with JQ1 at the same concentrations (BRYO 10/JQ1, ING 100/JQ1). PMA and ionomycin (PMA/IONO, 50ng/ml and 1uM respectively) were used as a positive control. (TIF) [file pone.0174516.s002.tif]

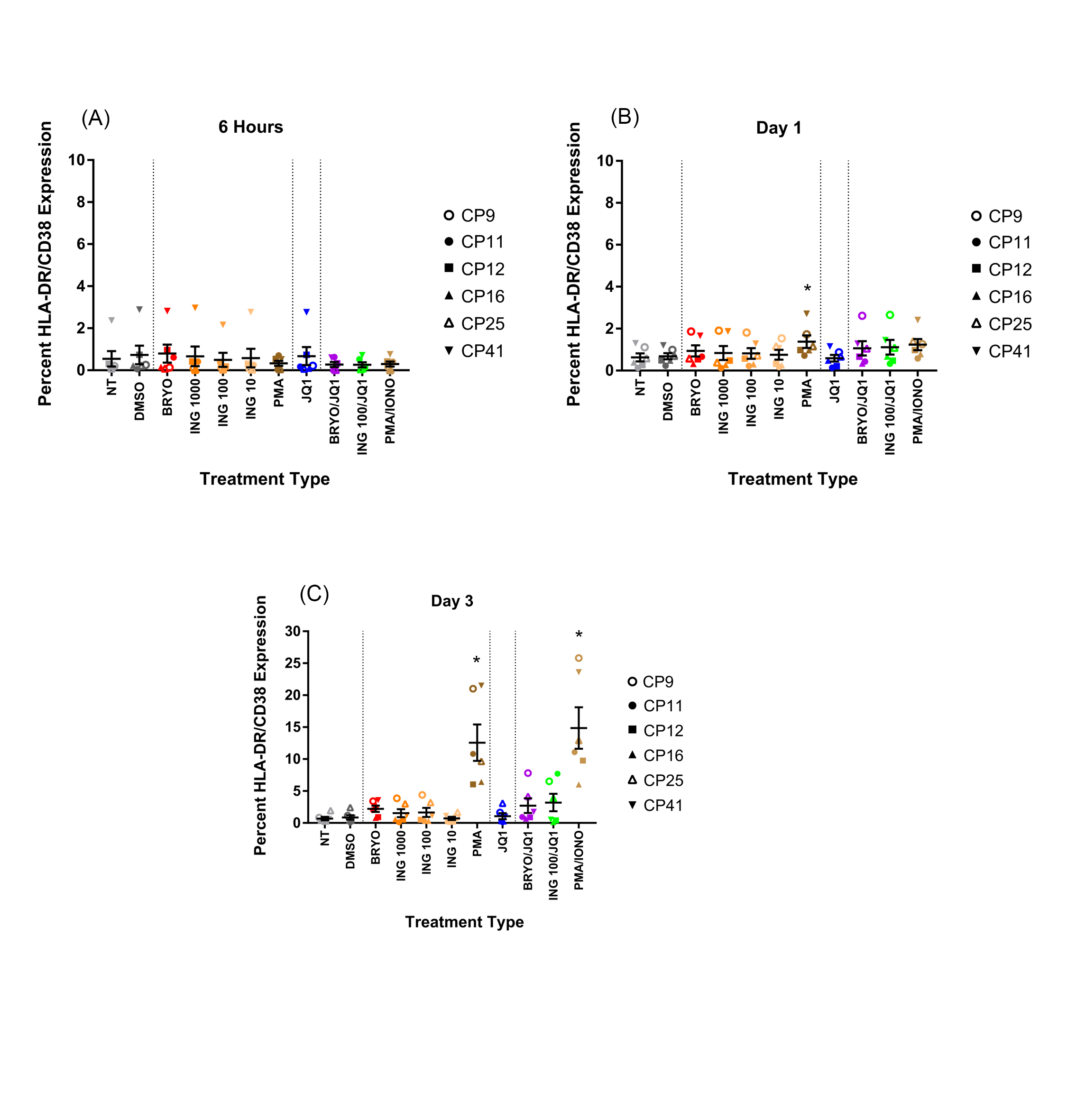

Supplement: S3 Fig — PBMCs from 6 CPs were treated with LRAs for 6 h, washed, then cultured for up to three days and examined for CD38 and HLA-DR co-expression on CD8 + T cells. Mean expression ± standard error is indicated for each treatment at (A) 6 hours, (B) day 1, and (C) day 3. Symbols directly above treatments indicate differences from the DMSO control (* p< 0.05). Cells were either non treated (NT) or treated with DMSO (0.1%), bryostatin-1 at 10nM (BRYO), ingenol-B at 1000, 100 or 10nM (ING 1000, ING 100, ING 10 respectively), PMA at 50ng/mL (PMA) and JQ1 at 1uM (JQ1). In some experiments the PKC-agonists were combined with JQ1 at the same concentrations (BRYO 10/JQ1, ING 100/JQ1). PMA and ionomycin (PMA/IONO, 50ng/ml and 1uM respectively) were used as a positive control. (TIF) [file pone.0174516.s003.tif]

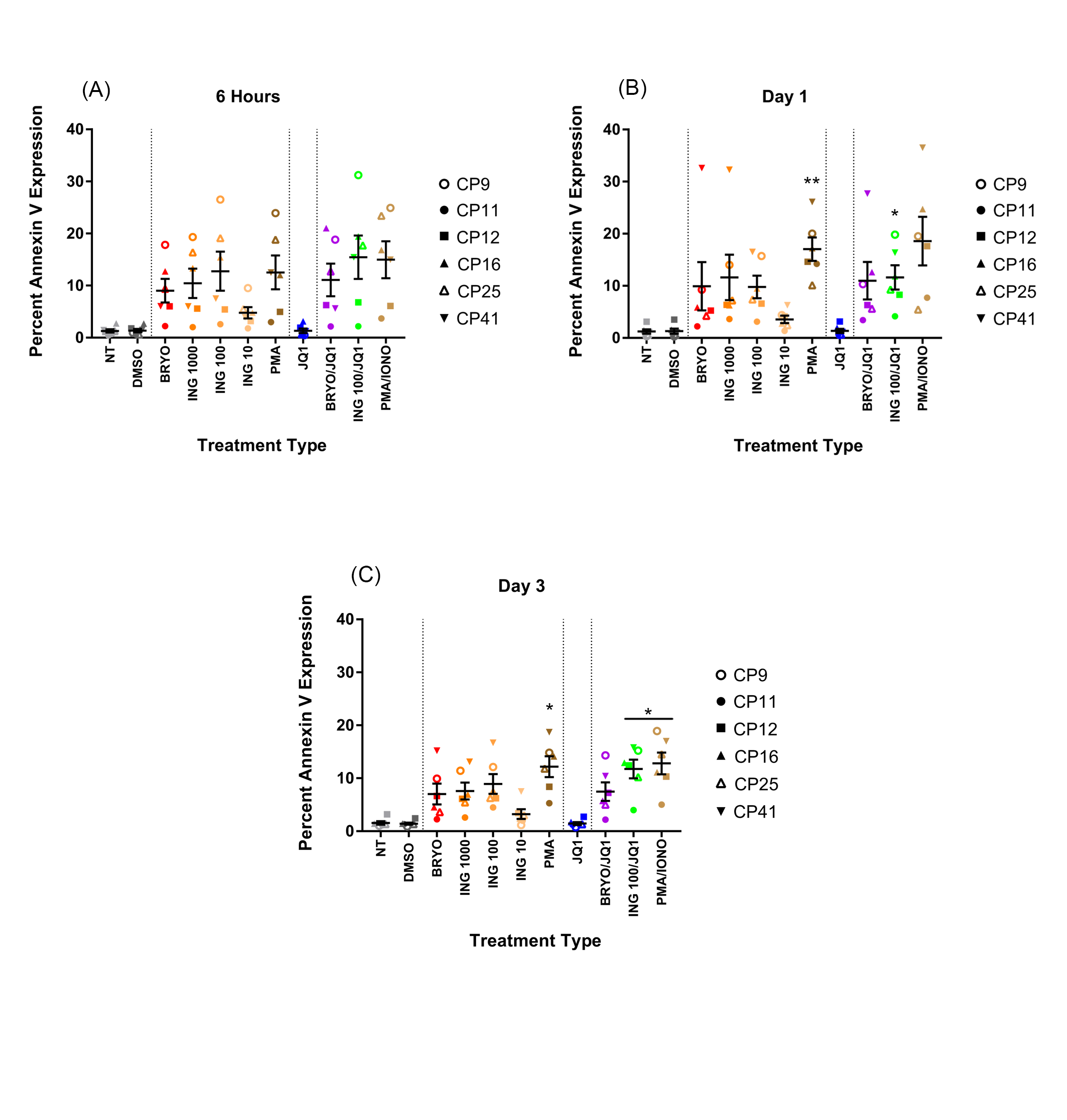

Supplement: S4 Fig — PBMCs from 6 CPs were isolated and treated with LRAs for 6 h, washed, then cultured for up to three days and examined for cell death as measured by percent annexin V expression. Mean expression ± standard error is indicated for each treatment at (A) 6 hours, (B) day 1, and (C) day 3. Symbols directly above treatments indicate differences from the DMSO control (* p< 0.05, ** p < 0.01). Cells were either non treated (NT) or treated with DMSO (0.1%), bryostatin-1 at 10 nM (BRYO), ingenol-B at 1000, 100 or 10nM (ING 1000, ING 100, ING 10 respectively), PMA at 50ng/mL (PMA) and JQ1 at 1uM (JQ1). In some experiments the PKC-agonists were combined with JQ1 at the same concentrations (BRYO 10/JQ1, ING 100/JQ1) or with PMA and ionomycin (PMA/IONO, 50ng/ml and 1uM respectively). (TIF) [file pone.0174516.s004.tif]

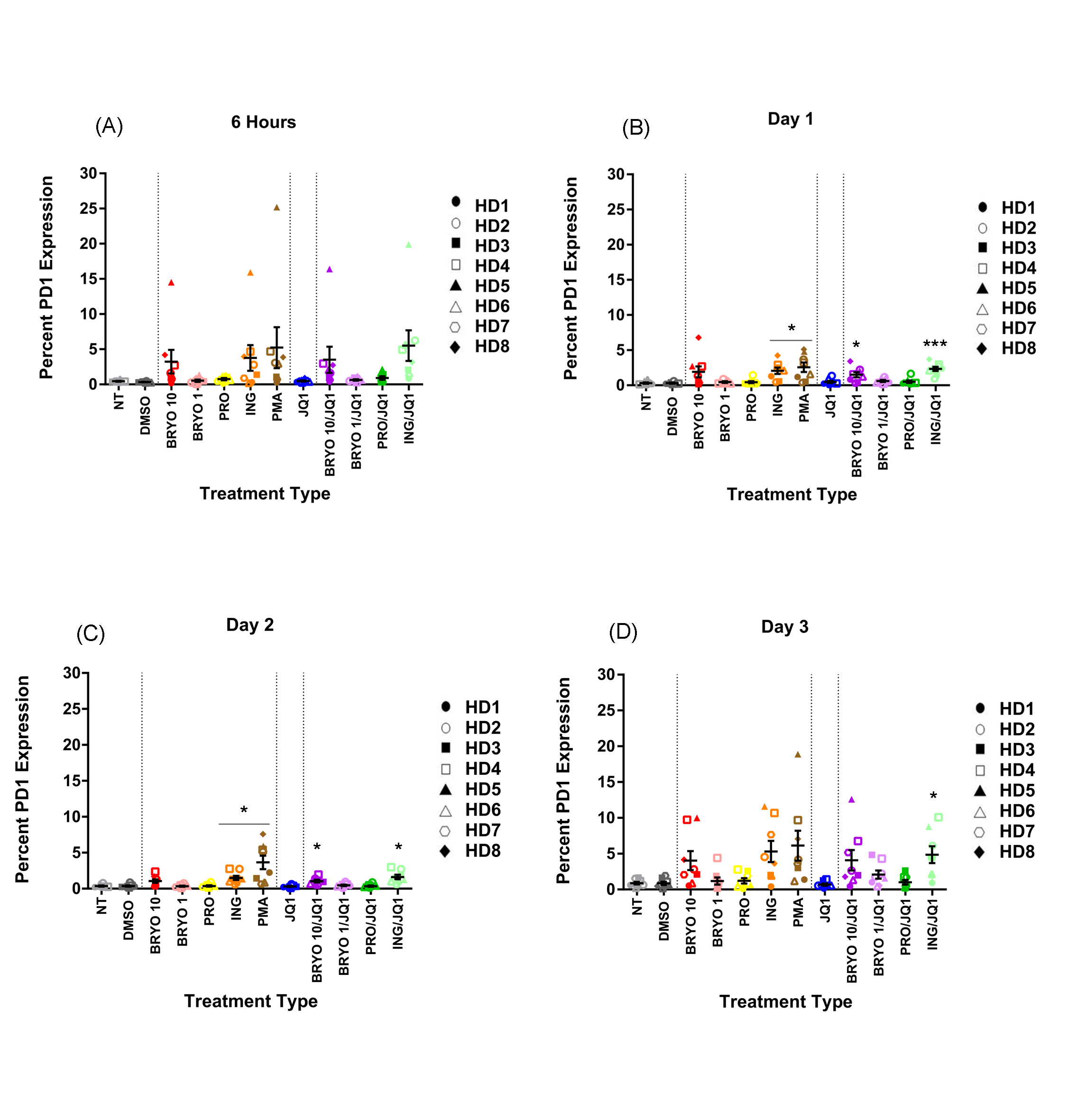

Supplement: S5 Fig — PBMCs from 8 HDs were treated with LRAs for 6 h before being washed and cultured for up to three days and examined for cell exhaustion as measured by percent PD1 expression. Mean expression ± standard error is indicated for each treatment at (A) 6 hours, (B) day 1, (C) day 2 and (D) day 3. Symbols directly above treatments indicate differences from the DMSO control. * p< 0.05, ** p < 0.01, *** p <0.001, **** p < 0.0001. Cells were either non treated (NT) or treated with DMSO (1%), bryostatin-1 at 10 or 1 nM (BRYO 10 and BRYO 1 respectively), prostratin at 0.3 uM (PRO), ingenol-B at 100nM (ING), PMA at 50ng/mL (PMA) and JQ1 at 1uM (JQ1). In some experiments the PKC agonists were combined with JQ1 at the same concentrations (BRYO 10/JQ1, BRYO 1/JQ1, PRO/JQ1, ING/JQ1). (TIF) [file pone.0174516.s005.tif]

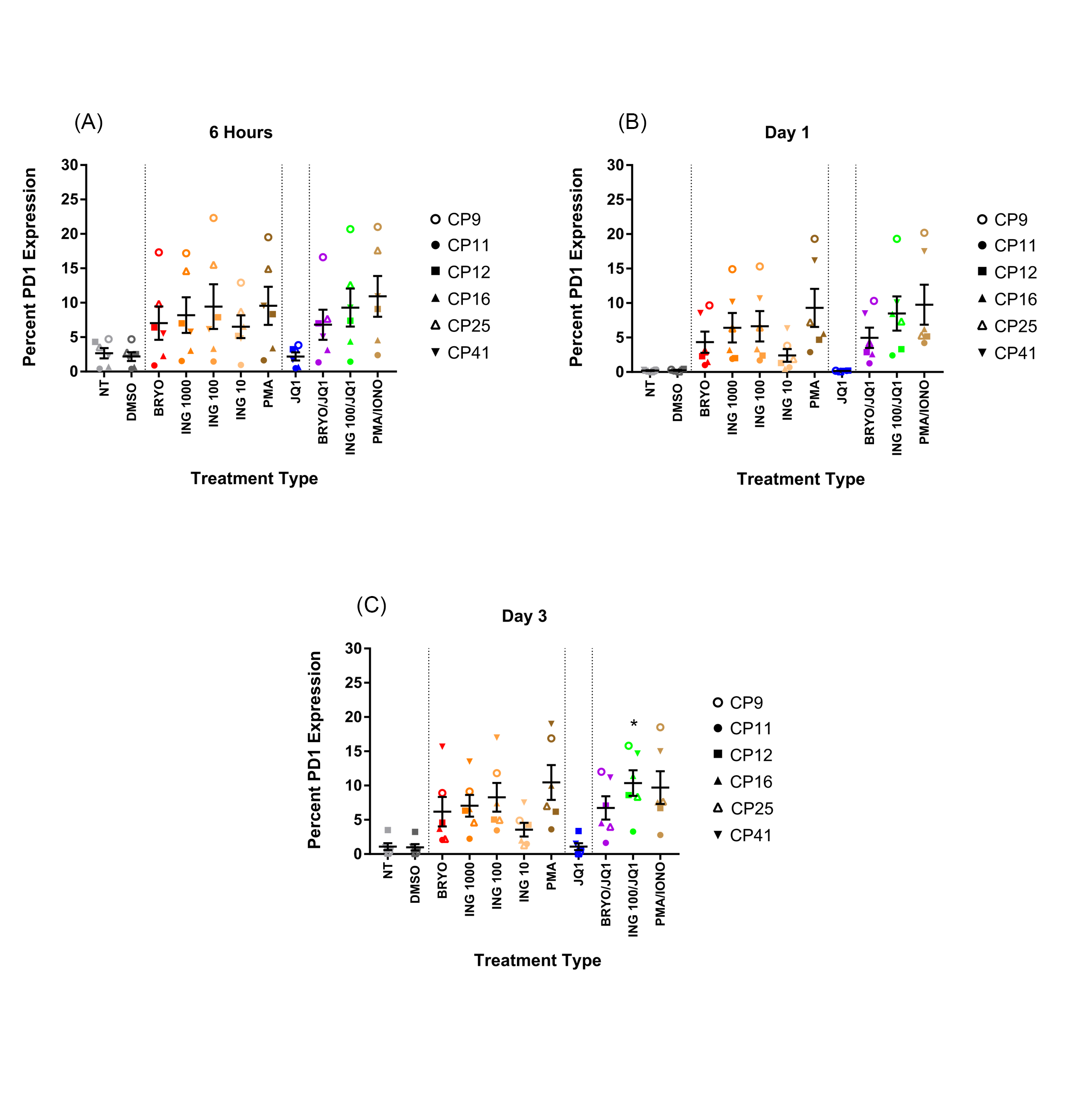

Supplement: S6 Fig — PBMCs from 6 CPs were isolated and treated with LRAs for 6 h, washed, then cultured for up to three days and examined for cell death as measured by percent annexin V expression. Mean expression ± standard error is indicated for each treatment at (A) 6 hours, (B) day 1, and (C) day 3. Symbols directly above treatments indicate differences from the DMSO control (* p< 0.05). Cells were either non treated (NT) or treated with DMSO (0.1%), bryostatin-1 at 10 nM (BRYO), ingenol-B at 1000, 100 or 10nM (ING 1000, ING 100, ING 10 respectively), PMA at 50ng/mL (PMA) and JQ1 at 1uM (JQ1). In some experiments the PKC-agonists were combined with JQ1 at the same concentrations (BRYO 10/JQ1, ING 100/JQ1) or with PMA and ionomycin (PMA/IONO, 50ng/ml and 1uM respectively). (TIF) [file pone.0174516.s006.tif]

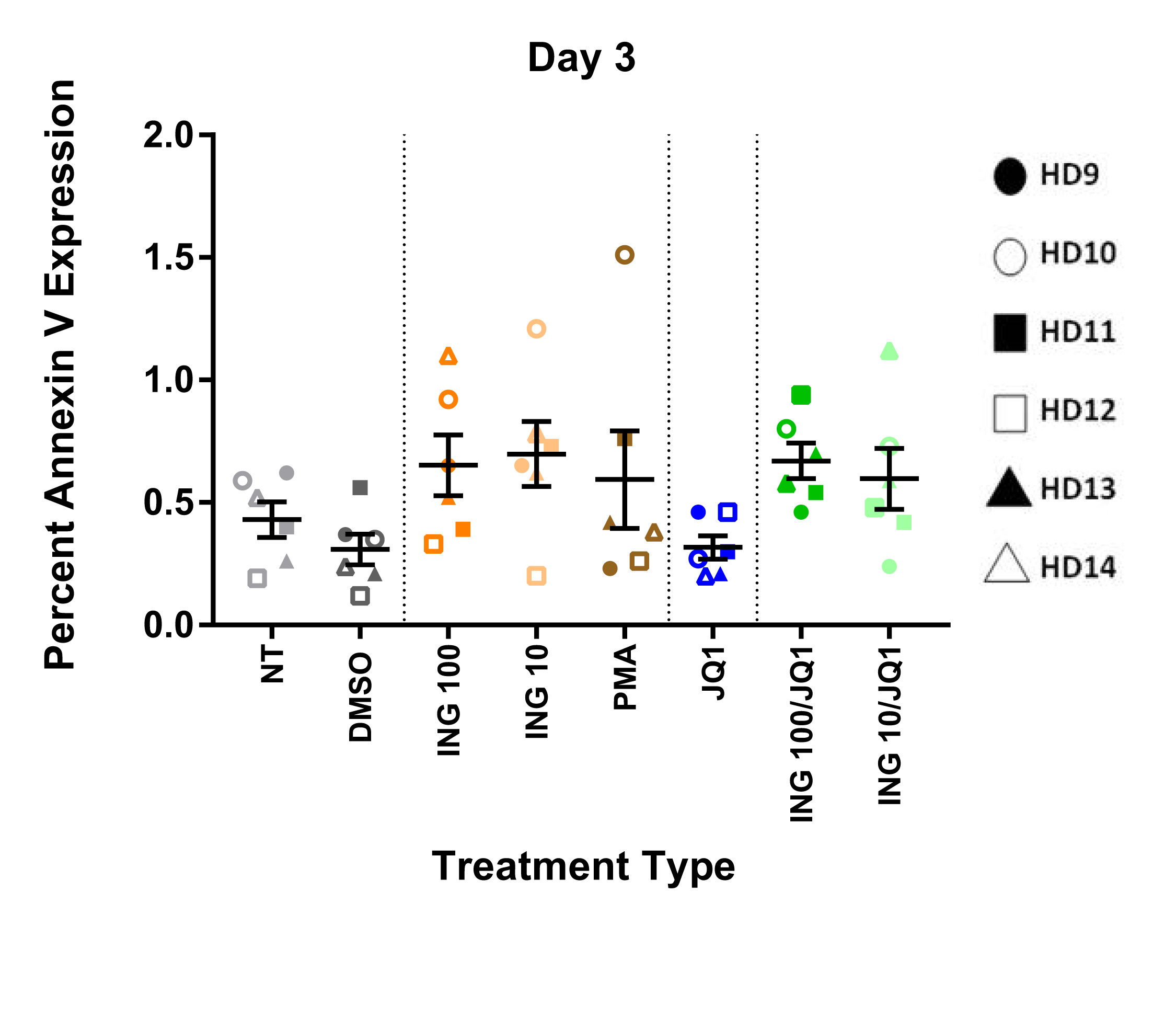

Supplement: S7 Fig — PBMCs were isolated from 6 healthy donors and CD4+ and CD8+ T cells enriched. Cells were combined at a 1:1 ratio, co-cultured and treated with LRAs for 6hrs after which cells were extensively washed and incubated for an additional 3 days before examining for CD8+ T cell death by percent annexin V expression. Mean expression ± standard error is indicated for each treatment at day 3. No significant differences in cell death were observed, relative to the DMSO control. Cells were either non treated (NT) or treated with DMSO (1%), ingenol-B at 100nM or 10nM (ING 100 or ING 10), PMA at 50ng/mL (PMA), JQ1 at 1uM (JQ1) and ingenol-B/JQ1 combinations at the same concentrations(ING 100/JQ1, ING 10/JQ1). (TIF) [file pone.0174516.s007.tif]

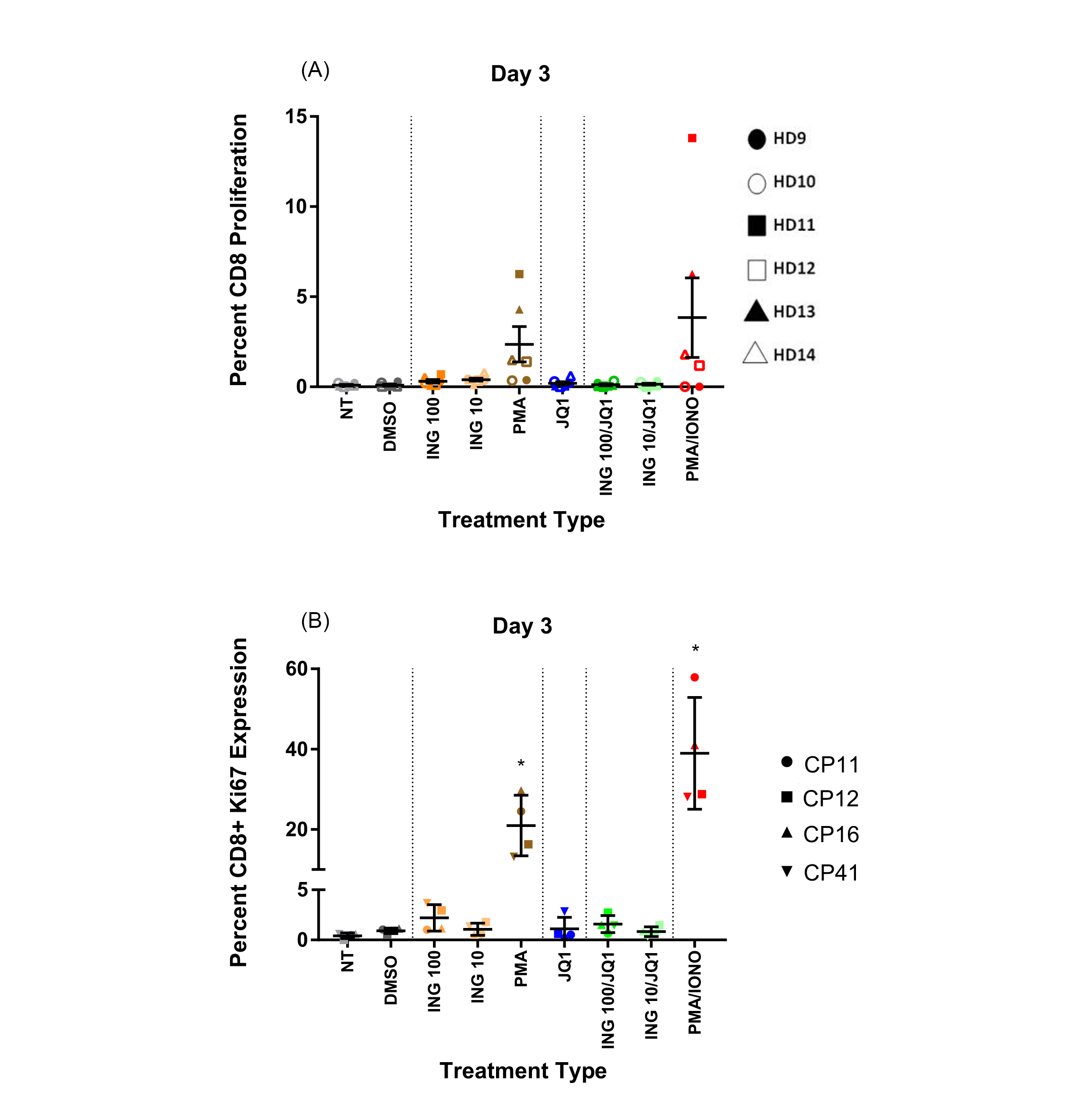

Supplement: S8 Fig — (A) PBMCs were isolated from 6 HD PBMCs and CD4+ and CD8+ T cells enriched. Cells were combined at a 1:1 ratio and exposed to CellTrace CFSE. They were treated with LRAs for 6hrs incubated for an additional 3 days before examination for CD8+ T cell proliferation by percent CFSE expression. Mean expression ± standard error is indicated for each treatment at day 3. Cells were either non treated (NT) or treated with DMSO (1%), ingenol-B at 100nM or 10nM (ING 100 or ING 10), PMA at 50ng/mL (PMA), PMA/Ionomycin (PMA/IONO, 50ng/ml and 1uM respectively), JQ1 at 1uM (JQ1) and ingenol-B/JQ1 combinations at the same concentrations(ING 100/JQ1, ING 10/JQ1). (B) PBMCs were isolated from 6 CPs and treated for 6 hours with LRAs. They were then extensively washed and incubated for an additional 3 days before intracellular staining with the Ki-67 monoclonal antibody. Mean expression ± standard error is indicated for each treatment. (TIF) [file pone.0174516.s008.tif]

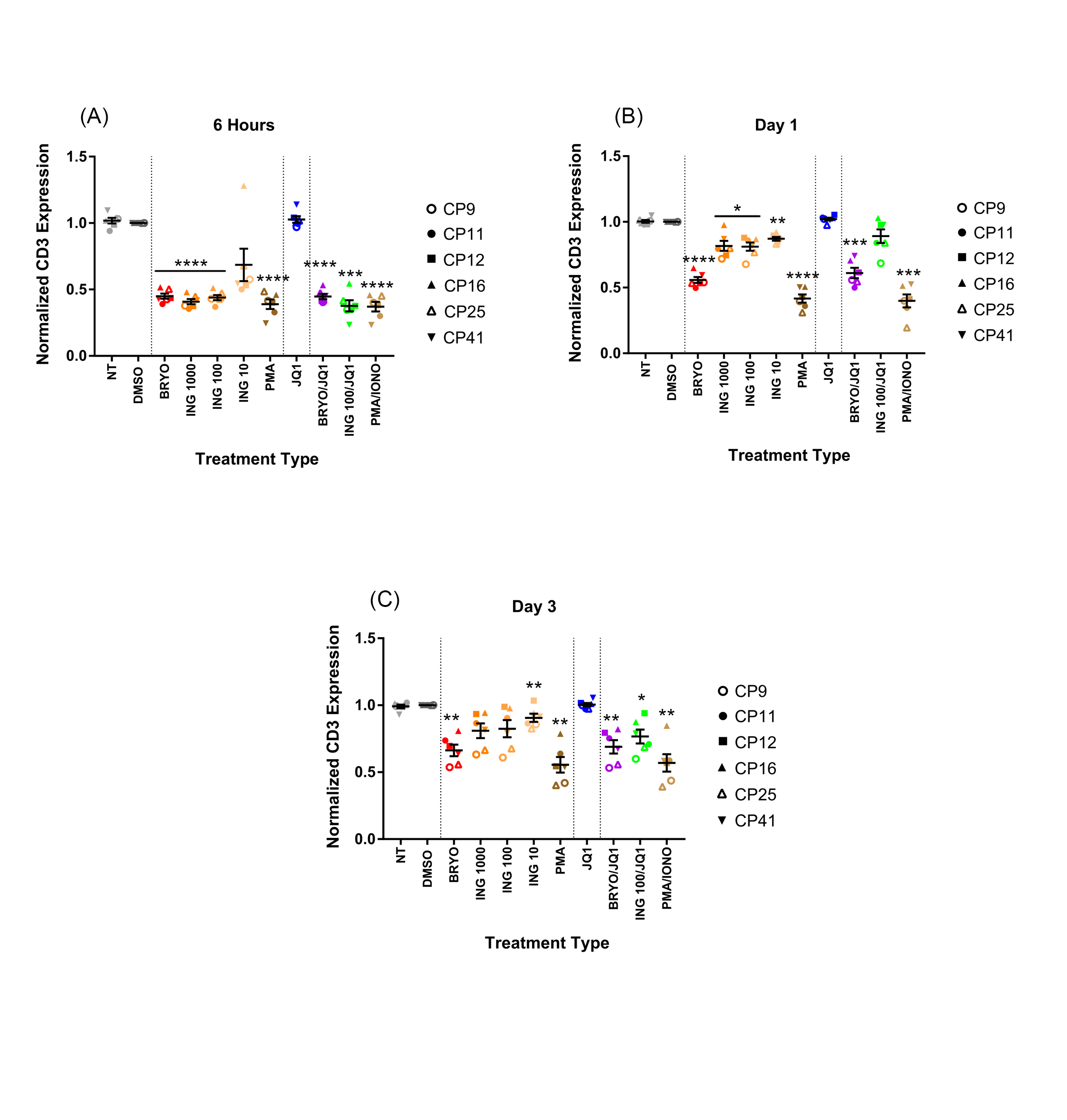

Supplement: S9 Fig — PBMCs from 6 CPs were isolated and treated with LRAs for 6 h, washed, then cultured for up to three days and examined for CD3 expression. Mean expression ± standard error is indicated for each treatment at (A) 6 hours, (B) day 1, and (C) day 3. Symbols directly above treatments indicate differences from the DMSO control (* p< 0.05, ** p < 0.01, *** p < 0.001, **** p < 0.0001). Cells were either non treated (NT) or treated with DMSO (0.1%), bryostatin-1 at 10 nM (BRYO), ingenol-B at 1000, 100, or 10nM (ING 1000, ING 100, ING 10 respectively), PMA at 50ng/mL (PMA) and JQ1 at 1uM (JQ1). In some experiments the PKC-agonists were combined with JQ1 at the same concentrations (BRYO 10/JQ1, ING 100/JQ1) or with PMA and ionomycin (PMA/IONO, 50ng/ml and 1uM respectively). (TIF) [file pone.0174516.s009.tif]
